# Attenuated African swine fever viruses and the live vaccine candidates: a comprehensive review

**DOI:** 10.1128/spectrum.03199-23

**Published:** 2024-10-08

**Authors:** Jiaqi Fan, Haisheng Yu, Faming Miao, Junnan Ke, Rongliang Hu

**Affiliations:** 1 College of Life Sciences, Ningxia University, Yinchuan, Ningxia, China; 2 Guangzhou Eighth People’s Hospital, Guangzhou Medical University, Guangzhou, China; 3 Key Laboratory of Prevention & Control for African Swine Fever and Other Major Pig Diseases, Ministry of Agriculture and Rural Affairs Changchun, Changchun, Jilin, China; 4 Changchun Veterinary Research Institute, Chinese Academy of Agricultural Sciences, Changchun, Jilin, China; Oklahoma State University College of Veterinary Medicine, Stillwater, Oklahoma, USA

**Keywords:** African swine fever virus, genetic deletion, live attenuated isolates, gene-modified viruses, vaccine candidates

## Abstract

The African swine fever virus (ASFV) is spreading worldwide and causing huge economic losses to the global pig industry. The ASFV genome is 170–193 kb in length, contains approximately 150 open reading frames, and encodes more than 200 proteins, most of which have unknown functions. Owing to the unique viral structure, replication strategy, large number of genes of unknown function, and complicated pathogenesis, vaccine development research is challenging. Several naturally attenuated ASFV isolates have been extensively investigated and many genetically manipulated, gene-deleted, and cell-adapted ASFVs have been reported. Currently, live attenuated viruses prepared from weakly virulent strains are an efficient method to provide effective protection in vaccinated pigs; however, these have seldom been widely approved for vaccine use, except in Vietnam. Herein, we summarize the attenuated isolates or vaccine candidates for live vaccines derived from different sources, including naturally mutated, attenuated, cell-adapted, and genetically modified recombinant ASFVs. This will help to understand the gene function and immunogenicity of attenuated live ASFV, as well as the shortcomings of these viruses as vaccine candidates, and provide clues to prepare live, efficient, and safe vaccines for African swine fever.

**IMPORTANCE:**

Outbreaks of African swine fever (ASF) have caused devastating losses to the global pig industry. Pigs immunized with ASFV attenuated virus can resist the lethal challenge of a strongly virulent virus. Here, we summarize the virulence of naturally mutated, cell-adapted, and genetically recombinant ASFV for pigs, and the protective effect after facing an attack challenge. We also analyze the advantages and disadvantages of ASFV attenuated viruses as vaccine candidates to provide clues for the preparation of efficient and safe live African swine fever vaccines.

## INTRODUCTION

African swine fever (ASF) is an acute, febrile, hemorrhagic disease caused by the African swine fever virus (ASFV). It is characterized by a short disease course, high fever, and hemorrhagic lesions, with a mortality rate of up to 100% from acute infections (1, 2). The occurrence of ASF in certain regions or countries must be notified to the World Organization for Animal Health (WOAH). ASFV is a nucleocytoplasmic large DNA virus, belonging to *Asfivirus*, family Asfarviridae (3). The ASFV is a double-stranded DNA virus with an icosahedral symmetric structure that replicates predominantly in the cytoplasm of infected cells. It has a genome length of 170–193 kb, contains approximately 150 open reading frames (ORFs), and encodes more than 200 proteins (4–6). The diversity in viral genome length and the number of ORFs is a typical feature of ASFV, which results from multiple gene family (MGF) insertions and deletions in the left variable region (LVR, 38–47 kb at the left end) and the right variable region (RVR, 13–16 kb at the right end) of the genome (5, 7, 8). The ASFV is classified into 24 genotypes based on the B646L (p72) gene sequence, all of which are transmitted in Africa. Genotypes I and II are transmitted across continents (9, 10). Since 2007, ASFV has been transmitted outward through Georgia, to the European Union in 2014, and to China in 2018, and the ASF epidemic has become a global concern (3, 11, 12).

**Fig 1:**
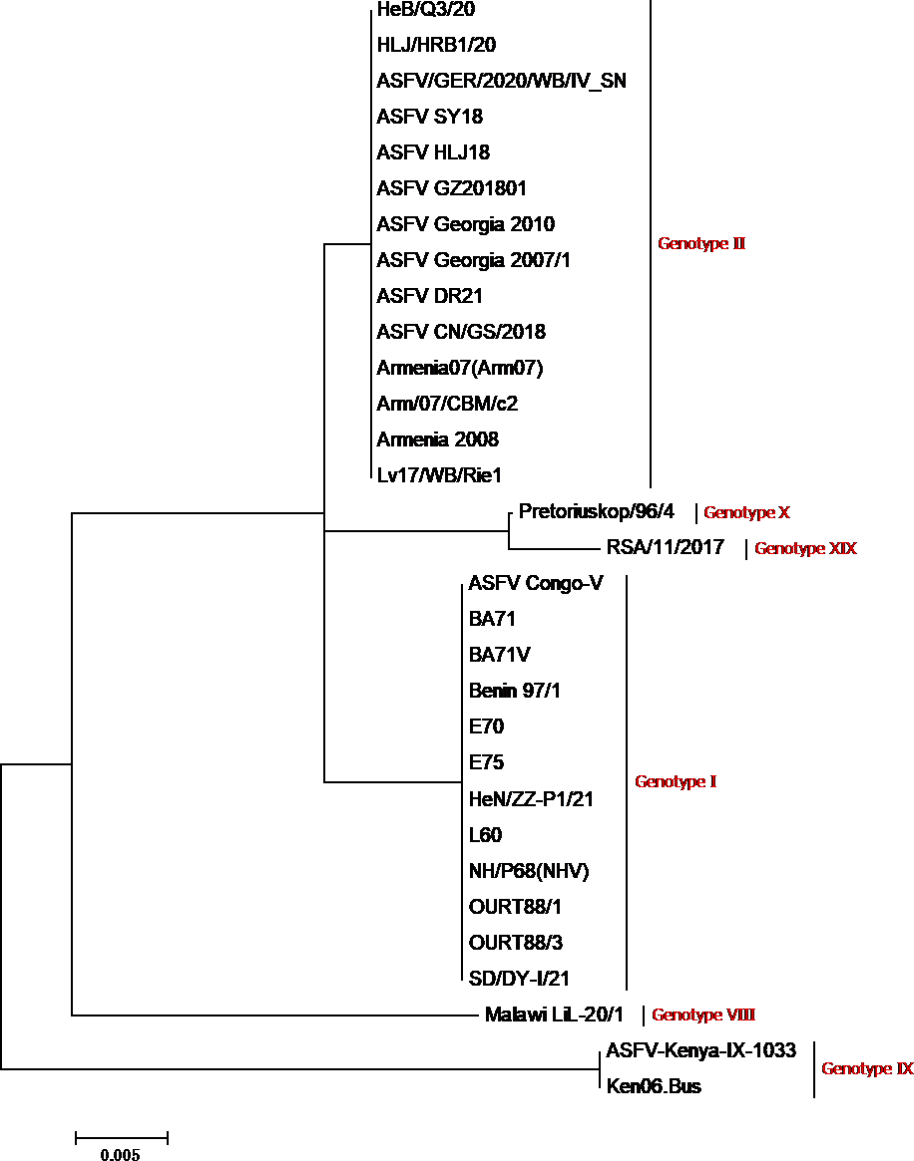
Evolutionary tree of different ASFV genotypes examined in the present study

Although ASF has been spreading for more than a hundred years since its discovery in Kenya in 1921, few vaccines have been approved. The inactivated ASFV virus has proven to be ineffective (13, 14). The live vaccine is considered the most promising ASF vaccine by professionals and government officials (15). Although multiple immunogenic ASFV proteins have been identified, subunit vaccines and DNA vaccine strategies based on single ASFV antigenic targets or multi-target cocktails that protect against ASFV have been inconsistent (16–19). Live attenuated viruses (LAVs) have been demonstrated to have great potential as vaccine strategies, providing 100% homologous protection and partial heterologous protection (20–22). Live attenuated viruses include naturally attenuated isolates, artificial gene-deficient strains, and cell-adapted attenuated or avirulent strains. Despite several studies demonstrating good protection with LAVs, the failure of field trials in Spain and Portugal, where immunization with attenuated ASFV strains caused widespread chronic ASF infection in pigs, leaves the use of LAVs to be considered. This review summarizes the natural attenuated, cell-adapted attenuated, and experimental recombinant strains of ASFV, compares the virulence and immunogenicity of the virus, and provides a theoretical basis for the screening of immunogenic antigens and the development of future vaccine candidates.The phylogenetic tree of the different African swine fever virus isolates involved in this study is shown in Fig. 1.

## RESULTS

### Naturally attenuated ASFV isolates

Years after the ASF epidemic in some regions, the mortality rates of pigs have been observed to decline over time, becoming subacute, chronic, or subclinical forms of the disease caused by the emergence of moderate and low-virulence virus isolates (23). Low-virulence natural mutants of ASFV isolated from soft ticks, chronically infected domestic pigs, and wild boars do not cause death in domestic pigs and are immunologically protective. Several naturally attenuated isolates have been reported. Among them, NH/P68 (genotype I), OURT88/3 (genotype I), and Lv17/WB/Rie1 (genotype II) were considered vaccine-candidate strains of ASFV. The virulence and protective efficacy of naturally attenuated ASFV isolates on pigs are shown in Table 1.

**TABLE 1:** Virulence and protective efficacy of low-virulence natural ASFV mutants on pigs

Virus strain and genotypes	Country of origin	Immunization dose (ON/IM/IN) and survival rate	Challenge dose and survival rate	Reference
OURT88/3, I	Portugal	10^4^ TCID_50_ (IM), 100% (12/12)	10^4^ HAD_50_, OURT88/1 (IM), 100% (12/12)	(24)
		10^3^ TCID_50_ (IN), 100% (6/6)	10^4^ TCID_50_, OURT88/1 (IM), 100% (6/6)	(25)
		10^4^ TCID_50_ (IN), 100% (6/6)	10^4^ TCID_50_, OURT88/1 (IM), 100% (6/6)	
		10^5^ TCID_50_ (IN), 100% (6/6)	10^4^ TCID_50_, OURT88/1 (IM), 66.7% (4/6)	
		10^3^ TCID_50_ (IM), 100% (6/6)	10^4^ TCID_50_, OURT88/1 (IM), 50% (3/6)	
		10^4^ TCID_50_ (IM), 100% (6/6)	10^4^ TCID_50_, OURT88/1 (IM), 66.7% (4/6)	
		10^5^ TCID_50_ (IM), 50% (3/6)	10^4^ TCID_50_, OURT88/1 (IM), 66.7% (2/3)	
NH/P68, I	Portugal	5 × 10^6^CPE_50_(ON/IM), 100% (31/31)	5 × 10^6^CPE_50_, L60 (IM), 100% (19/19)	(26)
		10^5^ TCID_50_ (IM), 100% (4/4)	10 HAD_50_, ASFV Armenia07 (IM), 75% (3/4)	(27)
		10^2^ TCID _50_ (IM), 100% (3/3)	10 HAD_50_, ASFV Armenia07 (IM), 33% (1/3)	
SD/DY-I/21, I	China	10^3^ TCID_50_ (IM), 83.3% (5/6)	/^*a*^	(28)
		10^6^ TCID_50_ (IM), 100% (6/6)	/^*a*^	
JS/LG/21, I	China	10^6^ HAD_50_ (IM), 0% (0/6)	/^*a*^	(2)
Lv17/WB/Rie1, II	Latvia	10 TCID_50_ (IM), 100% (2/2)	Co-cultured with pigs inoculated with a highly virulent strain (HAD-ASFV Lv2017/WB/Zieme24), Lv17/WB/Rie1-immunized pigs remained viable for a long time.	(29)
		10^4^ TCID_50_(ON), 100% (8/8)	10 HAD_50_ ,ASFV Armenia07 (IM)，100% (8/8)	(30)
		2 × 10^2^ FFU(IM), 60% (3/5)	10 HAD_50_, Armenia/07 (IM), 100% (3/3)	(31)
HLJ/HRB1/20, II	China	10^3^ TCID_50_ (IM), 100% (4/4)	/^*a*^	(32)
		10^6^ TCID_50_ (IM), 25% (1/4)	/^*a*^	
HeB/Q3/20, II	China	10^3^ TCID_50_ (IM), 100% (4/4)	/^*a*^	
		10^6^ TCID_50_ (IM), 50% (2/4)	/^*a*^	
ASFV-DR21, II	Dominican Republic	10^4^ HAD_50_ (IM), 50% (0/5)	/^*a*^	(33)
		10^4^ HAD_50_ (ON), 50% (2/4)	/^*a*^	

^*a*^ No challenge experiments were performed. FFU: fluorescent focus unit (FFU).

NH/P68, a low-virulence strain of ASFV isolated from a domestic pig with clinical signs of chronic ASF, and OURT88/3, isolated from a soft tick on a farm, are non-lethal and cause asymptomatic or chronic ASF lesions in domestic pigs, and the virus is non-hemadsorbing (non-HAD) (34, 35). Pigs immunized with OURT88/3 are resistant to the lethal challenge with OURT88/1; however, the efficiency of protection in pigs depends on the route and dose of OURT88/3 administration (24, 25). NHV of 5 × 10^6^ 50% cytopathic effect (CPE_50_) was inoculated into pigs by oral-nasal and intramuscular injection, and all pigs survived during the observation period. In total, 19 pigs showed no obvious clinical signs of ASF, 12 pigs showed chronic clinical signs of ASF, and all 19 pigs without obvious clinical signs of ASF survived the lethal challenge with L60 (5 × 10^6^CPE_50_) (26). In China, similar sequence identity and virulence but unknown sources of genotype I ASFV have also been reported. For example, SD/DY-I/21 and HeN/ZZ-P1/21 were isolated from two pig producer in China and were found to be highly similar to NH/P68 and OURT88/3 based on their phylogenetic tree, but showed significant differences in whole-genome sequence analysis (28). After immunization of pigs with SD/DY-I/21 at 10^3^ 50% tissue culture infective doses (TCID_50_) and 10^6^ TCID_50_ by intramuscular (IM) injection, all pigs survived during the observation period, except for one pig that died on day 16 (one pig in the 10^3^ TCID_50_ group) and showed distinct clinical signs of ASF (rash, skin necrosis, and arthritis) (28). The virus was detected in the blood of all pigs in the sentinel group cohabiting with the experimental group, demonstrating that SD/DY-I/21 is highly transmissible in pigs. JS/LG/21 is a highly transmissible intensely virulent genotype I ASFV isolated in China, but its genome is a chimeric recombination of genotypes I and II viruses ASFV (2).

Lv17/WB/Rie1, a non-HAD attenuated ASFV, was isolated from the Latvian wild boar (29). All pigs immunized with 10 TCID_50_ of Lv17/WB/Rie1 survived during the observation period, and when they cohabitated with highly virulent strain (HAD-ASFV Lv2017/WB/Zieme24) infected pigs, the virulent virus was isolated from the tonsils of Lv17/WB/Rie-immunized pigs but the pigs still survived for a long time (29). Eight wild boars were orally inoculated (ON) with 10^4^ TCID_50_ of Lv17/WB/Rie; all pigs survived during immunization observation and were resistant to lethal challenge with 10 50% hemadsorbing doses (HAD_50_) of ASFV Armenia07 (30). Subsequently, several genotype II ASFVs with incompletely attenuated virulence have been identified, including HLJ/HRB1/20, HeB/Q3/20, and ASFV DR21. HLJ/HRB1/20 and HeB/Q3/20 are low-virulence non-HAD ASFVs isolated in China that cause chronic or subacute diseases when infected at 10^3^ TCID_50_ (32). ASFV DR21, a virus found in the Dominican Republic, has a 50% survival rate when inoculated oronasally (ON), and the surviving animals exhibit a mild form of the disease (33). Notably, deletion of genes in naturally attenuated strains reduces viremia but may reduce immunogenicity and diminish the protective effect (36, 37). Although some natural, less virulent strains can protect against the challenge of virulent strains, they can cause chronic ASF infection in domestic pigs. Therefore, natural low-virulence strains are not suitable candidates for vaccine development.

### Cell-adapted ASFV strains

African swine fever virus can grow in primary cells such as peripheral blood leukocytes, peripheral blood monocytes, bone marrow (BM) cells, and alveolar macrophages in pigs (38). As the virus is present in a variety of organs in pigs, it can also grow in other primary cells derived from swine tissues, such as porcine kidney cells, hepatocytes, endothelial cells, and testicular cells (39). However, monocyte-macrophages are the main target cells for ASFV infection, and the WOAH recommends the use of bone marrow cells and monocytes for ASFV isolation tests. The limitation of ASFV for efficient replication in porcine primary macrophages is an important factor preventing the large-scale commercial production of LAVs.

There is a period of adaptation for the growth of ASFV in passaged cell lines. A portion of the virus gradually disappears after several generations of cell line cultivation, where another portion can grow in the cell line after genomic modification (40). Adapted cell lines for ASFV include pig kidney cell lines (SPEV), baby hamster kidney cells (BHK), African green monkey kidney cells (Vero/COS), Plum Island porcine epithelial cells (PIPEC), iPAM, IPKM, WSL, HEK293T, Zuckermann macrophage-4 (ZMAC-4), and pig macrophage cell lines (41–44). African swine fever virus undergoes significant genomic alterations during adaptation to cell lines, such as BA71V (BA71 infection of Vero cells), WSL-propagated ASFV-Kenya-IX-1033 (ASFV-Kenya-IX-1033 infection of WSL cells), Lv17/WB/Rie1-Δ24 (Lv17/WB/Rie1 infection of Cos7 cells), L60V (Lisbon 60 infection of Vero cells), Haiti/V(HT/V), Dominican Republic I/V and II/V(DR-I/V and DR-II/V, respectively), Brazil II/V (BRA-II/V), E70MS (E70 infection of MS monkey kidney cells), E75-CV1 (E75 infection of CV1 cells), ASFV Congo-a (ASFV Congo-V infection of SPEV and BM cells), VNUA-ASFV-LAVL2 (VNUA-ASFV-05L1 infection of PAM and 3D4/21 cells), and ASFV-P121 (ASFV-HLJ/18 infection of HEK293T cells) (40, 42, 45–51). However, an increase in the number of passages of ASFV cell-adapted strains is usually accompanied by a decrease in virulence (BA71V, ASFV Congo-a, ASFV-G/VP110, and VNUA-ASFV-LAVL2), which may be accompanied by a loss of protection (ASFV-G/VP110)(40, 47, 50). Notably, ASFV can not only be subjected to cell passages to achieve attenuated virulence but also to incomplete inactivation to obtain a weakly virulent strain. The laboratory of Ploufragan-Plouzané-Niort heated ASFV Georgia 2007/1 at 60°C for 2 hours and fortuitously generated an attenuated strain named ASFV-989. Compared to the parental virus, the ASFV-989 strain genome has a deletion of 7458 nucleotides located in the 5″-end encoding region of MGF 505/360 (52).

ASFV-G-ΔI177L/ΔLVR was passaged 30 times consecutively in PIPEC with no change in the genome, immunogenicity, and protective efficacy of the virus (53). ASFV-Kenya-IX-1033 was passaged more than 20 times in WSL and retained high virulence (54). Although ASFV can replicate efficiently in some cell lines (all viral characteristics remain unaltered), the number of viral transmissions into cells is relatively low. ASFV-G was passaged 30 times consecutively in Vero cells and the virulence of the virus started to decrease; at 110 passages, the virus completely lost virulence and failed to induce protection against parental viral challenge (40). Studies on ASFV adaptation in cell lines require long-term experimental validation. The virulence and protective effects of cell-adapted ASFV strains are shown in Table 2.

**TABLE 2:** Virulence and protective effect of cell passaged low-virulence ASFV on pigs

Parental virus	Cells and generation	Virus strain	Immunization dose (ON/IM/IN) and survival rate	Challenge dose and survival rate	Reference
ASFV-G-∆I177L	IPKM,10	ASFV-G-∆I177Lp10	10^3^ HAD_50_(IM),100% (5/5)	10^2^ HAD_50_, ASFV Georgia 2010(IM), 100% (5/5)	(44)
ASFV Georgia 2007/1	Vero, 30	ASFV-G/VP30	10^2^ HAD_50_(IM),60% (3/5)	/^*a*^	(40)
			10^4^ HAD_50_(IM), 0% (0/5)	/^*a*^	
	Vero,60	ASFV-G/VP60	10^2^ HAD_50_(IM),100% (5/5)	/^*a*^	
			10^4^ HAD_50_(IM),0% (0/5)	/^*a*^	
	Vero, 80	ASFV-G/VP80	10^2^ HAD_50_(IM),100% (5/5)	/^*a*^	
			10^4^ HAD_50_(IM), 0% (0/5)	/^*a*^	
	Vero, 110	ASFV-G/VP110	10^2^ HAD_50_(IM),100% (5/5)	/^*a*^	
			10^4^ HAD_50_(IM),100% (5/5)	10^4^ HAD_50_, ASFV Georgia 2007/1(IM), 0% (0/5)	
ASFV Congo-V (K49）	SPEV, 50 + BM, 262	ASFV Congo-a (KK262)	10^6^ TCID_50_ (IM), 100% (5/5)	10^4^ HAD_50_, ASFV Congo-V(IM), 100% (5/5)	(47)
Lv17/WB/Rie1	COS7, 8 + PAM, 3	Lv17/WB/Rie1-Δ24	2 × 10^2^ FFU(IM) + 2 × 10^4^ FFU(IM), 100% (5/5)	10^2^ HAD_50_, Armenia/07 (IM), 0% (0/5)	(51)
E75	CV-1, 5	E75-CV1	10^5^ TCID_50_ (IM), 100% (4/4)	10^2^ TCID_50,_ E75(IM),100% (4/4)	(49)
VNUA-ASFV-05L1	PAM, 65 + 3D4/21, 55	VNUA-ASFV-LAVL2	10^2^ HAD_50_(IM), 100% (5/5)	8 × 10^3^ HAD_50_, VNUA-ASFV-05L1(IM), 100% (5/5)	(50)
			10^3^ HAD_50_(IM), 100% (5/5)	8 × 10^3^ HAD_50_, VNUA-ASFV-05L1(IM), 100% (5/5)	
			10^4^ HAD_50_(IM), 100% (5/5)	/^*a*^	
			10^5^ HAD_50_(IM), 100% (5/5)	/^*a*^	
OURT 88/3	ZMAC-4, 12	OURT88/3 ZMAC-4	10^4^ TCID_50_ (IM), 100% (6/6)	10^4^ TCID_50_ (IM), OURT 88/1,100% (6/6)	(43)
ASFV Georgia 2007/1	Georgia 2007/1 strain incubated at 60°C for 2 h	ASFV-989	10^3^ HAD_50_(IM),100% (4/4)	10^3^ HAD_50_, ASFV Georgia 2007/1(IM), 100% (4/4)	(52)
			10^4^ HAD_50_(ON),100% (6/6)	10^3^ HAD_50_, ASFV Georgia 2007/1(IM), 100% (6/6)	
			10^4^ HAD_50_(ON),100% (6/6)	10^3^ HAD_50_, ASFV Georgia 2007/1(ON), 100% (6/6)	
			10^3^ HAD_50_(IM),100% (6/6)	10^3^ HAD_50_, ASFV Georgia 2007/1(IM), 83.3% (5/6) (challenge at 14 days)	
			10^4^ HAD_50_(ON),100% (6/6)	10^4^ HAD_50_, ASFV Georgia 2007/1(ON), 100% (6/6) (challenge at 14 days)	

^*a*^ No challenge experiments were performed.

### Attenuated ASFVs using genetic manipulation

Differences in the genomes of different ASFVs are enormous, and there are diverse variations in the sequences of ASFV genomes of the same or different genotypes, resulting in differences in the virulence of the virus. Gene-deficient ASFVs, created by deleting one or more specific genes in the ASFV genome, can significantly reduce virulence (21, 22, 55, 56). However, differences in the dose and route of immunization with recombinant viruses can lead to significant differences in the virulence and protection kinetics. African swine fever virus with excessive gene deletion may reduce immunogenicity and lack protective efficacy (57, 58).

### Recombinant ASFVs with multiple gene deletions

Initially, LAVs were studied based on the missing fragments in naturally attenuated ASFVs. The virulence of recombinant ASFV with multiple deletions is shown in Table 3. Therefore, more research has been conducted to develop multi-gene deleted ASFVs, most of which have focused on MGF and CD2V gene deletions. Recently, researchers have developed a variety of gene-deleted viruses with completely attenuate virulence and 100% protection against lethal challenges; for example, ArmΔCD2v-ΔA238L (59), ASFV-SY18-∆CD2v/UK (60), ASFV-G-∆I177L/∆EP402R (61), ASFV-ΔH240R-Δ7R (62), ASFV-ΔMGF110/360-9L (63), ASFV-ΔQP509L/QP383R (55), ASFV-ΔECM3, HLJ/18–6GD (56), ASFV-G-ΔMGF (64), HLJ/18–7GD (56), ASFV-GS-Δ18RΔNLΔUK (65), and ASFV-G-ΔI177L/ΔLVR (53). Thus, LAVs are potential vaccine candidates. Notably, ASFV with several deleted genes may have reduced immunogenicity and lack protective efficacy, such as ASFV-G-ΔMGF/ΔE184L (66), HLJ/18–9GL&UK-del (56), Lv17/WB/Rie1-ΔCD-ΔGL (51), ASFV-G-Δ9GL/ΔCD2v (67), ASFV-G-Δ9GL/ΔCD2v/ΔEP153R (67), ASFV-G-Δ9GL/ΔNL/ΔUK (68), BeninΔA179L (69). A booster immunization protocol was also explored, hoping that multiple intramuscular injections of ASFV would protect pigs from lethal parental or genotypic viruses. The effectiveness of protection against lethal challenge with virulent wt-type strains after multiple doses of immunization is shown in Table 4.

**TABLE 3:** ASFV recombinant viruses with multiple gene deletions

Gene	Virus strain	Recombinant virus	Immunization dose (ON/IM/IN) and survival rate	Challenge dose and survival rate	Reference
A104R (premature termination) & B125L	ASFV SY18	ASFV-SY18-ΔB125R/etA104R	10^2^ TCID_50_ (IM), 0% (0/5)	/^*a*^	(70)
			10^4^ TCID_50_ (IM), 0% (0/5)	/^*a*^	
A238L(MGF505-7R) & EP402R (CD2v)	ASFV-Kenya-IX-1033	ASFV-Ke-∆EP402R∆A238L	10^3^ TCID_50_ (IM), 100% (9/9)	10^2^ TCID_50_, ASFV-Kenya-IX-1033 (IM),50% (4/8)	(71)
	Arm/07/CBM/c2	ArmΔCD2v-ΔA238L	10^2^ TCID_50_ (IM), 100% (4/4)	10^2^ HAD_50_, ASFV/Korea/Pig/Paju/2019 (IM), 100% (4/4)	(59)
B119L(9Gl) & DP96R(UK)	ASFV HLJ/18	HLJ/18–9GL&UK-del	10^3^ TCID_50_ (IM), 100% (6/6)	200 PLD_50_, ASFV HLJ/18 (IM), 0% (0/6)	(56)
			10^5^ TCID_50_ (IM), 100% (6/6)	200 PLD_50_, ASFV HLJ/18 (IM), 0% (0/6)	
	ASFV Georgia 2007	ASFV-G-Δ9GL/ΔUK	10^2^ HAD_50_ (IM), 100% (9/9)	10^3^ HAD_50_, ASFV Georgia 2007 (IM), 44.4% (4/9)	(72)
			10^4^ HAD_50_ (IM), 100% (10/10)	10^3^ HAD_50_, ASFV Georgia 2007 (IM), 100% (10/10)	
			10^6^ HAD_50_ (IM), 100% (15/15)	10^3^ HAD_50_, ASFV Georgia 2007 (IM), 93.3% (14/15)	
DP71L(NL) & DP96R(UK)	OUR T88/3	OUR T88/3ΔDP2	10^4^ TCID_50_ (IM), 100% (6/6)	10^4^ HAD_50_, OUR T88/3 (IM), 66.6% (4/6)	(36)
DP96R(UK) & EP402R (CD2v)	ASFV Georgia 2010	ASFV-G-Δ9GL/ΔCD2v	10^3^ TCID_50_ (IM), 100% (4/4)	10^3^ TCID_50_, ASFV Georgia 2010 (IM),0% (0/4)	(67)
DP96R & EP402R (CD2v)	BA71	BA71∆CD2DP96R	10^6^ PFU (IM), 100% (5/5)	10^3^ GEC, ASFV Georgia 2007/1 (IM), 80% (4/5)	(73)
DP96R(UK)& EP402R (CD2v)	ASFV HLJ/18	HLJ/18-CD2v&UK-del	10^3^ TCID_50_ (IM), 50% (2/4)	/^*a*^	(56)
			10^5^ TCID_50_ (IM), 50% (2/4)	/^*a*^	
	ASFV-SY18	ASFV-SY18-∆CD2v/UK	10^4^ TCID_50_ (IM), 100% (5/5)	10^4^ TCID_50_, ASFV SY18 (IM), 100% (5/5)	(60)
EP153R & EP402R (CD2v)	BA71	BA71∆CD2EP153R	10^6^ PFU (IM), 100% (5/5)	10^3^ GEC, ASFV Georgia 2007/1 (IM), 60% (3/5)	(73)
EP402R & I177L	ASFV Georgia 2010	ASFV-G-∆I177L/∆EP402R	10^2^ TCID_50_ (IM), 100% (5/5)	10^2^ HAD_50_, ASFV Georgia 2010 (IM), 100% (5/5)	(61)
			10^6^ TCID_50_ (IM), 100% (5/5)	10^2^ HAD_50_, ASFV Georgia 2010 (IM), 100% (5/5)	
I177L & MGF110-5L-6L	ASFV Georgia 2010	ASFV-G-ΔI177L/ΔMGF110-5L-6L	10^6^ HAD_50_ (IM), 100% (5/5)	10^2^ TCID_50_, ASFV Georgia 2010 (IM), 40% (2/5)	(74)
H240R & MGF505-7R	ASFV HLJ/18	ASFV-ΔH240R-Δ7R	10^3^ HAD_50_ (IM), 100% (5/5)	10^2.5^ HAD_50_, ASFV HLJ/18(IM),100% (5/5)	(62)
			10^5^ HAD_50_ (IM), 100% (5/5)	10^2.5^ HAD_50_, ASFV HLJ/18(IM),100% (5/5)	
K145R & MGF 360–18R (DP148R)	ASFV Georgia 2007/1	Georgia∆K145R∆DP148R	10^3^ HAD_50_ (IM), 0% (0/4)	/^*a*^	(75)
MGF110-9L & MGF505-7R	ASFV CN/GS/2018	ASFV-Δ110–9L/505–7R	10^3^ HAD_50_ (IM), 100% (5/5)	10^2^ HAD_50_, ASFV GZ201801 (IM), 60% (3/5)	(76)
			10^4^ HAD_50_ (IM), 100% (5/5)	10^2^ HAD_50_, ASFV GZ201801 (IM), 83.3% (5/6)	
			10^5^ HAD_50_ (IM), 100% (5/5)	10^2^ HAD_50_, ASFV GZ201801 (IM), 100% (5/5)	
			10^6^ HAD_50_ (IM), 100% (5/5)	10^2^ HAD_50_, ASFV GZ201801 (IM), 100% (5/5)	
MGF110-9L & MGF360-9L	ASFV CN/GS/2018	ASFV-ΔMGF110/360-9L	10^4^ HAD_50_ (IM), 100% (6/6)	10^2^ HAD_50_, ASFV GZ201801 (IM), 100% (6/6)	(63)
MGF360-9L & MGF505-7R	ASFV CN/GS/2018	ASFV-Δ9L/Δ7R	10^2^ HAD_50_ (IM), 100% (6/6)	10^2^ HAD_50_, ASFV GZ201801 (IM), 83.3% (5/6)	(77)
QP509L & QP383R	ASFV CN/GS/2018	ASFV-ΔQP509L/QP383R	10^4^ HAD_50_ (IM), 100% (6/6)	10^2^ HAD_50_, ASFV GZ201801 (IM), 100% (6/6)	(55)
B119L(9Gl) & DP71L(NL) &DP96R(UK)	ASFV Georgia 2010	ASFV-G-Δ9GL/ΔNL/ΔUK	10^4^ HAD_50_ (IM), 100% (5/5)	10^2^ HAD_50_, ASFV Georgia 2010 (IM), 0% (0/5)	(68)
DP71L(NL) &DP96R(UK) & DP148R(MGF360-18R)	ASFV CN/GS/2018 (ASFV-GS)	ASFV-GS-Δ18RΔNLΔUK	10^4^ HAD_50_ (IM), 100% (5/5)	10^2^ HAD_50_, ASFV CN/GS/2018 (IM), 100% (5/5)	(65)
DP96R(UK) & EP153R & EP402R (CD2v)	ASFV Georgia 2010	ASFV-G-Δ9GL/ΔCD2v/ΔEP153R	10^3^ TCID_50_ (IM), 100% (4/4)	10^3^ TCID_50_, ASFV Georgia 2010 (IM),0% (0/4)	(67)
L7L & L8L & L9R & L10L & L11L	ASFV SY18	SY18△L7-11	10^3^ TCID_50_ (IM), 83.3% (5/6)	10^3^ TCID_50_, ASFV SY18 (IM), 100% (5/5)	(78)
			10^6^ TCID_50_ (IM), 100% (6/6)	10^3^ TCID_50_, ASFV SY18 (IM), 100% (6/6)	
MGF360-13L & MGF360-14L & MGF505-2R & MGF505-3R K145R	ASFV Georgia 2007/1 &	GeorgiaΔK145RΔMGF(B)	10^4^ HAD_50_ (IM), 0% (0/6)	/^*a*^	(79)
EP153R & EP402R (CD2v) & MGF360-12L & MGF360-13L & MGF360-14L	ASFV GZ201801	ASFV-ΔECM3	10^5^ TCID_50_ (IM), 100% (5/5)	10^2^ HAD_50_, ASFV GZ201801 (IM), 100% (5/5)	(80)
MGF505-1R & MGF505-2R& MGF505-3R & MGF360-12L & MGF360-13L & MGF360-14L	ASFV HLJ/18	HLJ/18–6GD	10^3^ TCID_50_ (IM), 100% (4/4)	200 PLD_50_, ASFV HLJ/18 (IM), 100% (4/4)	(56)
			10^5^ TCID_50_ (IM), 100% (4/4)	200 PLD_50_, ASFV HLJ/18 (IM), 100% (4/4)	
	ASFV Georgia 2007	ASFV-G-ΔMGF	10^2^ HAD_50_ (IM), 100% (10/10)	10^3^ HAD_50_, ASFV Georgia 2007 (IM), 100% (10/10)	(81)
			10^4^ HAD_50_ (IM), 100% (10/10)	10^3^ HAD_50_, ASFV Georgia 2007 (IM), 100% (10/10)	
			10^5^ HAD_50_ (ON), 100% (8/8)	10^4^ HAD_50_, ASFV/GER/2020/WB/IV_SN(IM), 100% (8/8)	(64)
MGF505-1R & MGF360-12L & MGF360-13L & MGF360-14L & MGF505-2R & MGF505-3R & CD2v	ASFV HLJ/18	HLJ/18–7GD	10^3^ TCID_50_ (IM), 100% (4/4)	200 PLD_50_, ASFV HLJ/18 (IM), 100% (4/4)	(56)
			10^5^ TCID_50_ (IM), 100% (4/4)	200 PLD_50_, ASFV HLJ/18 (IM), 100% (4/4)	
			10^6^ TCID_50_ (IM), 100% (5/5)	10^3^ HAD_50_, ASFV HLJ/18 (IM), 100% (5/5)	(2)
			10^6^ TCID_50_ (IM), 100% (5/5)	10^3^ HAD_50_, JS/LG/21 (IM), 0% (0/5)	
	JS/LG/21	JS/LG/21–7GD	10^6^ TCID_50_ (IM), 100% (10/10)	/^*a*^	(2)
	ASFV SY18	SY18ΔMGF/CD2v	10^4^ TCID_50_ (IM), 100% (4/4)	10^2.5^ TCID_50_, ASFV SY18 (IM), 60% (3/5)	(21)
MGF505-1R (L3FR) & MGF505-2R (L3NL) & MGF505-3R (L3QR) & MGF360-12L (L3HL) &MGF360-13L (L3IL) & MGF360-14L (L3LL) & DP71L(NL)	Malawi Lil-20/1	MalΔSVD	10^3^ TCID_50_ (IM), 100% (4/4)	/^*a*^	(82)
MGF505-1R & MGF360-12L & MGF360-13L & MGF360-14L & MGF505-2R & MGF505-3R & E184L	ASFV Georgia 2010	ASFV-G-ΔMGF/ΔE184L	10^4^ HAD_50_ (IM), 100% (5/5)	10^2^ HAD_50_, ASFV Georgia 2010 (IM), 0% (0/5)	(66)
			10^6^ HAD_50_ (IM), 100% (5/5)	10^2^ HAD_50_, ASFV Georgia 2010 (IM), 0% (0/5)	
MGF360-6L & MGF300-1L & MGF300-2R & MGF300-4L & MGF360-8L & MGF360-9L & MGF360-10L & MGF360-4L & MGF360-11L & I177L	ASFV Georgia 2010	ASFV-G-ΔI177L/ΔLVR	10^2^ HAD_50_ (IM), 100% (5/5)	10^2^ HAD_50_, ASFV Georgia 2010 (IM), 100% (5/5)	(53)
			10^4^ HAD_50_ (IM), 100% (5/5)	10^2^ HAD_50_, ASFV Georgia 2010 (IM), 100% (5/5)	
			10^6^ HAD_50_ (IM), 100% (5/5)	10^2^ HAD_50_, ASFV Georgia 2010 (IM), 100% (5/5)	
MGF360-10L & MGF360-11L & MGF360-12L & MGF360-13L & MGF360-14L & MGF505-1R & MGF505-2R & MGF505-3R (MGF360-10L, 11L, 12L, 13L, 14L and MGF530/505-1R, 2R, 3R)	Benin 97/1	BeninΔMGF	10^2^ TCID_50_ (IM), 100% (6/6)	10^4^ TCID_50_, Benin 97/1 (IM), 50% (3/6)	(58)
			10^3^ TCID_50_ (IM), 100% (6/6)	10^4^ TCID_50_, Benin 97/1 (IM), 66.7% (4/6)	
			10^4^ TCID_50_ (IM), 100% (6/6)	10^4^ TCID_50_, Benin 97/1 (IM), 83.3% (5/6)	
			10^3^ TCID_50_ (IN), 100% (6/6)	10^4^ TCID_50_, Benin 97/1 (IM), 66.7% (4/6)	
MGF300-4L & MGF360-8L & MGF360-9L & MGF360-10L & MGF360-11L & MGF505-1R & MGF360-12L & MGF360-13L & MGF360-14L & MGF505-2R & MGF505-3R	Armenia2007	ASFV Arm07ΔMGF	10^5^ TCID_50_ (IM), 100% (6/6)	/^*a*^	83
			10^7^ TCID_50_ (IM), 100% (6/6)	/^*a*^	
			10^3^ TCID_50_ (IM), 100% (3/3)	10^2^ HAD_50_, Armenia2007 (IM), 33.3% (1/3)	
			10^5^ TCID_50_ (IM), 100% (3/3)	10^2^ HAD_50_, Armenia2007 (IM), 66.7% (2/3)	
			10^7^ TCID_50_ (IM), 100% (6/6)	/^*a*^	
			10^3^ TCID_50_ (IM), 100% (3/3)	10^2^ HAD_50_, Armenia2007 (IM), 33.3% (1/3)	
			10^5^ TCID_50_ (IM), 100% (3/3)	10^2^ HAD_50_, Armenia2007 (IM), 66.7% (2/3)	

^*a*^ No challenge experiments were performed. Plaque-forming units (PFU), 50% pig lethal dose (PLD50), gene equivalent copies (GEC).

**TABLE 4:** Immunization regimen and protection efficacy of deletant ASFV^*a*^

Immunization regiment (ON/IM/IN) and survival rate	Challenge dose and survival rate	Reference
10^3^ TCID_50_ BeninΔA179L (IM) + 10^4^ TCID_50_ BeninΔA179L (IM) + 10^4^ TCID_50_ BeninΔA179L (IM),100%(4/4)	10^4^ TCID_50_, Benin 97/1 (IM),0% (0/4)	(69)
10^6^ TCID_50_ NH/P68ΔA238L (IM) + 10^5^ HAD_50_ L60 (IM), 100% (4/4)	10 HAD_50,_ Arm07 (IM), 0% (0/2)	(27)
10^6^ TCID_50_ NH/P68ΔA224L (IM)+ 10^5^ HAD_50_ L60 (IM), 100% (4/4)	10 HAD_50,_ Arm07 (IM), 50% (1/2)	(27)
10^6^ TCID_50_ NH/P68ΔEP153R (IM) + 10^5^ HAD_50_ L60 (IM), 100% (4/4)	10 HAD_50,_ Arm07 (IM), 0% (0/2)	(27)
10^6^ TCID_50_ NH/P68 (IM) + 10^5^ HAD_50_ L60 (IM), 100% (4/4)	10 HAD_50,_ Arm07 (IM), 100% (2/2)	(27)
3.3 × 10^4^ PFU BA71ΔCD2v (IM) + 3.3 × 10^4^ PFU BA71ΔCD2v (IM), 100% (4/4)	10^2^ HAU, Ken06.Bus (IM), 50% (2/4)	(84)
3.3 × 10^4^ PFU BA71ΔCD2v (IM) + 10^3^ HAU BA71 (IM), 100% (4/4)	10^2^ HAU, Ken06.Bus (IM), 100% (4/4)	(84)
2 × 10^6^ HAU ASFV Congo-a(IM) + 2 × 10^6^ HAU ASFV Congo-a(IM), 100% (6/6)	10^2^ HAU, ASFV Congo-V (IM), 100% (6/6)	(85)
10^3^ HAD_50_ BeninΔDP148R (IM) + 10^3^ HAD_50_ BeninΔDP148R (IM), 100% (6/6)	10^4^ HAD_50_, Benin 97/1 (IM),100% (6/6)	(86)
10^3^ HAD_50_ BeninΔDP148R (IN) + 10^3^ HAD_50_ BeninΔDP148R (IN), 100% (6/6)	10^4^ HAD_50_, Benin 97/1 (IM),83.3% (5/6)	(86)
2 × 10^2^ FFU Lv17/WB/Rie1/ΔCD-ΔGL(IM)【Lv17/WB/Rie1 deletes B119L & EP402R】+2 × 10^4^ FFU Lv17/WB/Rie1/ΔCD-ΔGL(IM) , 100% (5/5)	10^2^ HAD_50_, Armenia/07 (IM), 0% (0/5)	(51)
10^4^ HAD_50_ ASFV-G-∆MGF (IM) + 10^4^ HAD_50_ ASFV-G-∆MGF (IM), 100% (5/5)	10^4^ HAD_50_, Armenia08 (IM),100% (5/5)	(64)
10^3^ HAD_50_ ASFV-G-∆MGF (IM) + 10^3^ HAD_50_ ASFV-G-∆MGF (IM), 100% (5/5)	10^5^ HAD_50_, Armenia08 (IM),100% (5/5)	(64)
10^3^ TCID_50_ ASFV Arm07ΔMGF (IM) + 10^3^ TCID_50_ ASFV Arm07ΔMGF (IM), 100% (3/3)	10^2^ HAD_50_, Armenia2007 (IM), 66.7% (2/3)	(83)
10^5^ TCID_50_ ASFV Arm07ΔMGF (IM) + 10^5^ TCID_50_ ASFV Arm07ΔMGF (IM), 100% (3/3)	10^2^ HAD_50_, Armenia2007 (IM), 100% (3/3)	(83)
10^3^ TCID_50_ BeninΔDP148RΔEP402R(IM)+ 10^4^ TCID_50_ BeninΔDP148RΔEP402R(IM), 75% (3/4)	10^4^ HAD_50_, Benin 97/1 (IM),100% (3/3)	(87)
10^5^ TCID_50_ BeninΔDP148R (IM) + 10^5^ TCIAD_50_ BeninΔDP148R (IM), 100% (6/6)	10^4^ HAD_50_, Benin 97/1 (IM),100% (6/6)	(87)
10^5^ TCID_50_ BeninΔDP148RΔEP153R (IM) + 10^5^ TCID_50_ BeninΔDP148RΔEP153R (IM), 100% (6/6)	10^3^ HAD_50_, Benin 97/1 (IM),100% (6/6)	(87)
10^2^ HAD_50_ ASFV-Δ110–9L/505–7R (IM) + 10^2^ HAD_50_ ASFV-Δ110–9L/505–7R (IM), 100% (5/5)	10^2^ HAD_50_, ASFV GZ201801 (IM), 80% (4/5)	(76)
10^3^ HAD_50_ ASFV-Δ110–9L/505–7R (IM) + 10^3^ HAD_50_ ASFV-Δ110–9L/505–7R (IM), 100% (5/5)	10^2^ HAD_50_, ASFV GZ201801 (IM), 60% (4/5)	(76)
10^5^ TCID_50_ ASFV-GZΔI177L (IM) + 10^5^ TCID_50_ ASFV-GZΔI177L(IM), 100% (5/5)	10^4^ TCID_50_, ASFV GZ201801 (IM), 100% (5/5)	(88)
10^5^ TCID_50_ ASFV-GZΔI177LΔCD2v (IM) + 10^5^ TCID_50_ ASFV-GZΔI177LΔCD2v (IM), 100% (5/5)	10^4^ TCID_50_, ASFV GZ201801 (IM), 100% (5/5)	(88)
10^5^ TCID_50_ ASFV-GZΔI177LΔCD2vΔMGF (IM) + 10^5^ TCID_50_ ASFV-GZΔI177LΔCD2vΔMGF (IM), 100% (5/5)	10^4^ TCID_50_, ASFV GZ201801 (IM), 100% (5/5)	(88)
10^4^ HAD_50_ GeorgiaΔK145RΔMGF360-12LΔMGF505-1R (IM)【ASFV Georgia 2007/1 deletes K145R & MGF505-1R & MGF360-12L】+ 10^5^ HAD_50_ GeorgiaΔK145RΔMGF360-12LΔMGF505-1R (IM), 100% (6/6)	10^3^ HAD_50_, ASFV Georgia 2007/1 (IM), 33.3% (2/6)	(79)
10^4^ HAD_50_ GeorgiaΔMGF (IM)【ASFV Georgia 2007/1 deletes MGF360-9L & MGF360-10L & MGF360-11L & MGF360-12L & MGF360-13L & MGF360-14L & MGF505-1R, & MGF505-2R & MGF505-3R. & MGF505-4R.】+ 10^4^ HAD_50_ GeorgiaΔMGF (IM), 100% (8/8)	10^4^ HAD_50_, ASFV Georgia 2007/1 (IM), 25% (2/8)	(79)
10^4^ HAD_50_ GeorgiaΔK145RΔMGF(A) (IM)【ASFV Georgia 2007/1 deletes K145R & MGF505-1R & MGF360-12L & MGF360-13L & MGF360-14L】+ 10^4^ HAD_50_ GeorgiaΔK145RΔMGF(A) (IM) + 10^5^ HAD_50_ GeorgiaΔK145RΔMGF(A) (IM), 100% (6/6)	10^3^ HAD_50_, ASFV Georgia 2007/1 (IM), 66.7% (4/6)	(79)
10^4^ TCID_50_ BeninΔDP148RΔEP153RΔEP402R (IM) + 10^4^ TCID_50_ BeninΔDP148RΔEP153RΔEP402R (IM) + 10^6^ TCID_50_ BeninΔDP148RΔEP153RΔEP402R (IM), 100% (8/8)	10^3^ HAD_50_, Benin 97/1 (IM),75% (6/8)	(87)

^*a*^ Hemadsorbing unit (HAU).

### Single gene-deleted ASFV

Genetic manipulation of ASFVs has undergone a trend from multiple gene deletions to single-gene deletions. Borca et al. demonstrated that deletion of the I177L gene in the highly virulent strain ASFV-G could reduce the virulence of ASFV, induce a specific immune response, and resist lethal challenges with parental viruses (22). The search for virulence genes in the stable region of the ASFV genome and the construction of recombinant ASFV viruses with single-gene deletions have become a popular trend. Here, we summarize some of the virulence-associated genes identified in recent studies, whose deletion leads to reduced virulence in highly virulent ASFV. The virulence and protection efficiency of recombinant ASFV with a single-gene deletion are shown in Table 5.

**TABLE 5:** ASFV recombinant viruses with single gene deletion

Gene	Virus strain	Recombinant virus	Immunization dose (ON/IM/IN) and survival rate	Challenge dose and survival rate	Reference
A104R	ASFV Georgia 2010	ASFV-G-ΔA104R	10^2^ HAD_50_ (IM), 80% (4/5)	10^2^ HAD_50_, ASFV Georgia 2010 (IM), 0% (0/4)	(89)
A137R	ASFV Georgia 2010	ASFV-G-ΔA137R	10^2^ HAD_50_ (IM), 100% (5/5)	10^2^ HAD_50_, ASFV Georgia 2010 (IM), 100% (5/5)	(90)
A151R	ASFV Georgia 2010	ASFV-G-ΔA151R	10^2^ HAD_50_ (IM), 80% (4/5)	10^2^ HAD_50_, ASFV Georgia 2010 (IM), 75% (3/4)	(91)
A224L	NH/P68	NH/P68ΔA224L	10^6^ TCID_50_ (IM), 100% (4/4)	10^5^ HAD_50_, L60 (IM), 100% (4/4)	(27)
	Malawi Lil-20/1	Δ4CL	10^2^ TCID_50_ (IM), 0% (0/4)	/^*a*^	(92)
A238L (5EL)	NH/P68	NH/P68ΔA238L	10^6^ TCID_50_ (IM), 100% (4/4)	10^5^ HAD_50_, L60 (IM), 100% (4/4)	(27)
			10^2^ TCID_50_ (IM), 100% (5/5)	10 HAD_50_, ASFV Armenia07 (IM), 40% (2/5)	
	ASFV-Kenya-IX-1033	ASFV-Ke-∆A238	10^2^ TCID_50_ (IM), 66.7% (2/3)	/^*a*^	(71)
	ASFV E70	E70ΔA238L	10^5^ HAD_50_ (IM), 0% (0/2)	/^*a*^	(93)
			10^6^ HAD_50_ (IM), 0% (0/2)	/^*a*^	
	Malawi Lil-20/1	Δ5EL	10^2^ TCID_50_ (IM), 0% (0/4)	/^*a*^	(94)
A859L	ASFV Georgia 2007/1	ASFV-G-∆A859L	10^2^ HAD_50_ (IM), 0% (0/5)	/^*a*^	(95)
B119L(9Gl)	ASFV Georgia 2007/1	ASFV-G-Δ9GL	10^2^ HAD_50_ (IM), 100% (10/10)	10^3^ HAD_50_, ASFV Georgia 2007/1 (IM), 40% (2/5) (challenge at 21 days)	(96)
				10^3^ HAD_50_, ASFV Georgia 2007/1 (IM), 100% (5/5) (challenge at 28 days)	
			10^3^ HAD_50_ (IM), 100% (10/10)	10^3^ HAD_50_, ASFV Georgia 2007/1 (IM), 100% (5/5) (challenge at 21 days)	
				10^3^ HAD_50_, ASFV Georgia 2007/1 (IM), 100% (5/5) (challenge at 28 days)	
			10^4^ HAD_50_ (IM), 0% (0/5)	/^*a*^	
	Malawi Lil-20/1	Δ9GL	10^2^ TCID_50_ (IM), 100% (4/4)	10^4^ TCID_50_, Malawi Lil-20/1 (IM), 100% (4/4)	(97)
			10^4^ TCID_50_ (IM), 100% (4/4)	10^4^ TCID_50_, Malawi Lil-20/1 (IM), 100% (4/4)	
			10^6^ TCID_50_ (IM), 100% (4/4)	10^4^ TCID_50_, Malawi Lil-20/1 (IM), 100% (4/4)	
	Pretoriuskop/96/4	Pr4Δ9GL	10^4^ TCID_50_ (IM), 100% (4/4)	10^4^ TCID_50_, Pretoriuskop/96/4 (IM), 100% (4/4)	(16)
C84L	ASFV SY18	SY18ΔC84L	10^2^ TCID_50_ (IM), 100% (2/5)	/^*a*^	(98)
			10^5^ TCID_50_ (IM), 100% (1/5)	/^*a*^	
C962R	ASFV Georgia 2007	ASFV-G-ΔC962R	10^2^ HAD_50_ (IM), 0% (0/5)	/^*a*^	(99)
DP71L(NL)	ASFV Georgia 2010	ASFV-G-ΔNL	10^4^ HAD_50_ (IM), 80% (4/5)	/^*a*^	(68)
	ASFV E70	E70/43	10^2^ TCID_50_ (IM), 100% (4/4)	10^2^ TCID_50_, E70 (IM), 100% (4/4)	(100)
			10^3^ TCID_50_ (IM), 100% (5/5)	10^2^ TCID_50_, E70 (IM), 100% (5/5)	
	Malawi Lil-20/1	Mal-∆NL	10^2^ TCID_50_ (IM), 0% (0/5)	/^*a*^	(101)
	Pretoriuskop/96/4	Pr4∆NL	10^2^ TCID_50_ (IM), 14.3% (1/7)	/^*a*^	
DP96R(UK)	ASFV Georgia 2010	ASFV-G-ΔUK	10^4^ HAD_50_ (IM), 0% (0/5)	/^*a*^	(68)
	ASFV E70	ΔUK	10^2^ TCID_50_ (IM), 100% (12/12)	/^*a*^	(102)
E66L	ASFV Georgia 2010	ASFV-G-∆E66L	10^2^ HAD_50_ (IM), 0% (0/5)	/^*a*^	(103)
E111R	ASFV SY18	SY18-∆E111R	10^2^ TCID_50_ (IM), 40% (2/5)	/^*a*^	(104)
			10^5^ TCID_50_ (IM), 0% (0/5)	/^*a*^	
E165R	ASFV Georgia 2010	ASFV-G-∆E165R	10^2^ HAD_50_ (IM), 0% (0/5)	/^*a*^	(105)
E184L	ASFV Georgia 2010	ASFV-G-∆E184L	10^2^ HAD_50_ (IM), 60% (3/5)	10^2^ HAD_50_, ASFV Georgia 2010 (IM), 100% (3/3)	(66)
EP153R (8CR)	NH/P68	NH/P68ΔEP153R	10^6^ TCID_50_ (IM), 100% (4/4)	10^5^ HAD_50_, L60 (IM), 100% (4/4)	(27)
	Malawi Lil-20/1	Δ8CR	10^2^ TCID_50_ (IM), 0% (0/4)	/^*a*^	(106)
EP402R (CD2v/8DR)	Malawi Lil-20/1	Δ8-DR	10^2^ TCID_50_ (IM), 0% (0/8)	/^*a*^	(107)
	ASFV HLJ/18	HLJ/18-CD2v-del	10^3^ TCID_50_ (IM), 25% (1/4)	/^*a*^	(56)
			10^5^ TCID_50_ (IM), 50% (2/4)	/^*a*^	
	BA71	BA71∆CD2v	10^6^ PFU (IM), 100% (5/5)	10^3^ GEC, ASFV Georgia 2007/1 (IM), 100% (5/5)	(73)
			10^6^ PFU (IM), 100% (6/6)	field-harvested ticks naturally infected with the RSA/11/2017 virus, 83.3% (5/6)	(84)
			3.3 × 10^4^ PFU (IM), 100% (6/6)	10^2^ HAU, Ken06.Bus (IM), 16.6% (1/6)	
			10^6^ PFU (IM), 100% (6/6)	10^2^ HAU, Ken06.Bus (IM), 33.3% (2/6)	
	ASFV-Kenya-IX-1033	ASFV-Kenya-IX-1033-∆CD2v	10^3^ TCID_50_ (IM), 100% (9/9)	10^2^ HAD_50_ (IM), ASFV-Kenya-IX-1033 (IM), 87.5% (7/8)	(108)
	ASFV Congo-a (KK262)	ΔCongoCD2v	10^6^ TCID_50_ (IM), 100% (7/7)	10^4^ HAD_50_, ASFV Congo-V (IM),0% (0/7)	(47)
	ASFV Georgia2010	ASFV-G-Δ8DR	10^3^ TCID_50_ (IM), 0% (0/4)	/^*a*^	(109)
			10^4^ TCID_50_ (IM), 0% (0/6)	/^*a*^	
EP296R	ASFV Georgia 2010	ASFV-G-∆EP296R	10^2^ HAD_50_ (IM), 0% (0/5)	/^*a*^	(110)
H108R	ASFV Georgia 2007/1	ASFV-G-ΔH108R	10^2^ HAD_50_ (IM), 80% (4/5)	10^2^ HAD_50_, ASFV Georgia 2007/1 (IM), 100% (4/4)	(111)
H240R	ASFV HLJ/18	ASFV-∆H240R	10^3^ HAD _50_ (IM), 100% (6/6)	/^*a*^	(112)
			10^5^ HAD_50_ (IM), 83.3% (5/6)	/^*a*^	
	ASFV Georgia2010	ASFV-G-∆H240R	10^2^ HAD _50_ (IM), 40% (2/5)	10^2^ HAD_50_, ASFV Georgia2010 (IM), 100% (2/2)	(113)
I8L(L8L)	ASFV Georgia 2010	ASFV-G-ΔI8L	10^2^ HAD_50_ (IM), 0% (0/5)	/^*a*^	(114)
	ASFV SY18	SY18ΔL8L	10^3^ TCID_50_ (IM), 0% (0/4)	/^*a*^	(115)
I73R	ASFV GZ201801	ASFV-GZΔI73R	10^3^ TCID_50_ (IM), 100% (5/5)	10^4^ TCID_50_, ASFV GZ201801 (IM), 100% (3/3)	(116)
			10^5^ TCID_50_ (IM), 100% (5/5)	/^*a*^	
I177L	ASFV Georgia 2010	ASFV-G-ΔI177L	10^2^ HAD_50_ (IM), 100% (5/5)	10^2^ HAD_50_, ASFV Georgia 2010 (IM), 100% (5/5)	(22)
			10^6^ HAD_50_ (IM), 100% (5/5)	10^2^ HAD_50_, ASFV Georgia 2010 (IM), 100% (5/5)	
			2 × 10^6^ HAD_50_ (ON), 100% (5/5)	10^2^ HAD_50_, ASFV Georgia 2010 (IM), 100% (5/5)	(117)
			10^1^ HAD_50_ (IM), 100% (5/5)	10^2^ HAD_50_, TTKN/ASFV/DN/2019 (IM), 40% (2/5)	(118)
			10^2^ HAD_50_ (IM), 100% (5/5)	10^2^ HAD_50_, TTKN/ASFV/DN/2019 (IM), 100% (5/5)	
			10^3^ HAD_50_ (IM), 100% (5/5)	10^2^ HAD_50_, TTKN/ASFV/DN/2019 (IM), 100% (5/5)	
			10^4^ HAD_50_ (IM), 100% (5/5)	10^2^ HAD_50_, TTKN/ASFV/DN/2019 (IM), 100% (5/5)	
I226R	ASFV SY18	SY18ΔI226R	10^4^ TCID_50_ (IM), 100% (5/5)	10^2.5^ TCID_50_, ASFV SY18 (IM), 100% (5/5)	(21)
			10^7^ TCID_50_ (IM), 100% (5/5)	10^4^ TCID_50_, ASFV SY18 (IM), 100% (5/5)	
I267L	ASFV SY18	SY18ΔI267L	10^2^ TCID_50_ (IM), 0% (0/5)	/^*a*^	(119)
			10^5^ TCID_50_ (IM), 0% (0/5)	/^*a*^	
	ASFV CN/GS/2018	ASFVΔI267L	10 HAD_50_ (IM), 83.3% (5/6)	/^*a*^	(120)
I329L	OURT88/3	OURT88/3ΔI329L	10^4^ TCID_50_ (IM), 100% (6/6)	10^4^ TCID_50_, OURT88/1 (IM), 33.3% (2/6)	(37)
	Georgia 2007/1	GeorgiaΔI329L	10^4^ TCID_50_ (IM), 0% (0/6)	/^*a*^	
KP177R	ASFV Georgia 2010	ASFV-G-∆KP177R	10^2^ HAD_50_ (IM), 0% (0/5)	/^*a*^	(121)
K196R	ASFV-G/VP30	ASFV-G/V-ΔTK	10^4^ TCID_50_ (IM), 100% (4/4)	10^3^ HAD_50_, ASFV Georgia 2007/1 (IM), 0% (0/4)	(122)
			10^6^ TCID_50_ (IM), 100% (4/4)	10^3^ HAD_50_, ASFV Georgia 2007/1 (IM), 0% (0/4)	
	Malawi LiL-20/1	ASFV v5.3	10^4^ TCID_50_ (IM), 75% (3/4)	10^4^ TCID_50_, Malawi (IM), 66.7% (2/3)	(123)
L7L	ASFV SY18	SY18ΔL7L	10^3^ TCID_50_ (IM), 25% (1/4)	/^*a*^	(115)
L9R	ASFV SY18	SY18ΔL9R	10^3^ TCID_50_ (IM), 0% (0/4)	/^*a*^	(115)
L10L	ASFV SY18	SY18ΔL10L	10^3^ TCID_50_ (IM), 0% (0/4)	/^*a*^	(115)
L11L	Malawi LiL-20/1	Δl11L	10^2^ HAD_50_ (IM), 0% (0/3)	/^*a*^	(124)
	ASFV SY18	SY18ΔL11L	10^3^ TCID_50_ (IM), 100% (4/4)	10^3^ TCID_50_, ASFV SY18 (IM), 100% (4/4)	(115)
			10^6^ TCID_50_ (IM), 50% (2/4)	10^3^ TCID_50_, ASFV SY18 (IM), 100% (2/2)	
L60L	ASFV SY18	SY18ΔL60L	10^5^ TCID_50_ (IM), 60% (3/5)	10^2^ TCID_50_, ASFV SY18 (IM), 100% (3/3)	(125)
L83L	ASFV Georgia 2007	ASFV-G-ΔL83L	10^2^ HAD_50_ (IM), 0% (0/4)	/^*a*^	(126)
			10^3^ HAD_50_ (IM), 0% (0/4)	/^*a*^	
MGF100-1R	ASFV GZ201801	ASFV△MGF100-1R	10^4^ TCID_50_ (IM), 0% (0/3)	/^*a*^	(127)
MGF110-1L	ASFV Georgia 2007	ASFV-G-ΔMGF110-1L	10^2^ HAD_50_ (IM), 0% (0/5)	/^*a*^	(128)
MGF110-5L-6L	ASFV Georgia 2010	ASFV-G-ΔMGF110-5L-6L	10^2^ HAD_50_ (IM), 0% (0/5)	/^*a*^	(74)
MGF110-9L	ASFV CN/GS/2018	ASFV-Δ9L	10 HAD_50_ (IM), 60% (3/5)	/^*a*^	(129)
MGF 110–11L	Lv17/WB/Rie1	Lv17/WB/Rie1/d110-11L	2 × 10^2^ FFU(IM), 100%(5/5)	10 HAD_50_, Armenia/07 (IM), 100% (5/5)	(31)
MGF300-2R	ASFV HLJ/18	Del2R	10^2^ TCID_50_ (IM), 100% (4/4)	/^*a*^	(130)
			10^3^ TCID_50_ (IM), 50% (2/4)	/^*a*^	
MGF300-4L	ASFV HLJ/18	Del4L	10^2^ TCID_50_ (IM), 50% (2/4)	/^*a*^	(131)
MGF360-1L	ASFV Georgia 2007	ASFV-G-ΔMGF360-1L	10^2^ HAD_50_ (IM), 0% (0/5)	/^*a*^	(132)
MGF 360–9L	ASFV CN/GS/2018	ASFV-Δ360–9L	1 HAD_50_ (IM), 80% (4/5)	/^*a*^	(133)
MGF360-10L	ASFV CN/GS/2018	ASFV-Δ10L	10 HAD_50_ (IM), 100% (5/5)	/^*a*^	(134)
MFG360-15R(A276R)	NH/P68	NHV/P68ΔA276R	10^2^ TCID_50_ (IM), 100% (5/5)	10 HAD_50_, Arm07 (IM), 0% (0/5)	(27)
MGF360-16R	ASFV Georgia 2007	ASFV-G-ΔMGF360-16R	10^2^ HAD_50_ (IM), 0% (0/5)	/^*a*^	(135)
MGF 360–18R (DP148R)	Benin 97/1	BeninΔDP148R	10^3^ HAD_50_ (IM), 100% (5/5)	10^4^ HAD_50_ (IM), Benin 97/1 (IM),100% (5/5)	(86)
	ASFV Georgia 2007/1	Georgia∆DP148R	10^4^ HAD_50_ (IM), 0% (0/4)	/^*a*^	(75)
	ASFV HLJ/18	HLJ/18-DP148R-del	10^3^ TCID_50_ (IM), 0% (0/3)	/^*a*^	(56)
			10^5^ TCID_50_ (IM), 0% (0/3)	/^*a*^	
MGF-505–7R	ASFV CN/GS/2018	ASFV-ΔMGF-505–7R	10 HAD_50_ (IM), 100% (5/5)	/^*a*^	(136)
O174L	ASFV Georgia 2010	ASFV-G-∆O174L	10^2^ HAD_50_ (IM), 0% (0/5)	/^*a*^	(137)
QP509L	ASFV Georgia 2010	ASFV-G-∆QP509L	10^2^ HAD_50_ (IM), 0% (0/5)	/^*a*^	(138)
X69R	ASFV Georgia 2007/1	ASFV-G-ΔX69R	10^2^ HAD_50_ (IM), 0% (0/5)	/^*a*^	(139)

^*a*^ No challenge experiments were performed.

Currently, there are only five genetic deletions in which ASFV virulence is completely attenuated: A137R (90), I226R (21), I177L (22), I73R (116), MGF-360–10L (134), and MGF-505–7R (136). Borca et al. constructed a recombinant virus with the I177L gene deletion (ASFV-G-ΔI177L); pigs inoculated with either high (10^6^ HAD_50_) or low (10^2^ HAD_50_) doses survived and were resistant to challenge with the virulent strains (22). The A137R protein (pA137R or p11.5) inhibits type I interferon expression through autophagy-mediated lysosomal degradation of TBK1 (140). Gladue et al. constructed a deletant (ASFV-G-∆A137R) lacking the A137R gene that showed complete attenuation in pigs and could protect against the parental virus challenge (90). Zhang et al. had developed a recombinant virus (SY18∆I226R) with deletion of the I226R gene, which showed no virulence and was resistant to challenge with the parental strain even when inoculated in pigs at high doses (10^7^TCID_50_) (21). Liu et al. constructed the virus with I73R deletion (ASFV-GZΔI73R) and pigs inoculated with 10^3^TCID_50_ ASFV-GZΔI73R were resistant to challenge by the parental strain (116). All pigs inoculated with 10 HAD_50_ ASFV-ΔMGF-505–7R or ASFV-Δ10L survived during the observation period (134, 136). ASFV-ΔMGF-505–7R and ASFV-Δ10L were not used in the challenge experiment. Therefore, only ASFV-G-ΔI177L, ASFV-G-∆A137R, SY18∆I226R, and ASFV-GZΔI73R are suitable as vaccine candidates.

Some genetically deleted viruses are not completely attenuated; however, their safety is increased, as was observed in E184L (66), H108R (111), H240R (112), and L60L (125). Pigs infected with these viruses were 100% resistant to a lethal challenges with the parental strain. Notably, some gene-deficient viruses caused the expression of high ASFV-specific antibodies, but were not 100% protective (or completely unprotective) against the parental strain; examples include A104R (89), A151R (91), and K196R (123). A104R is a viral histone-like protein that regulates viral DNA topology and may be involved in viral DNA replication, transcription, and packaging (141). Pigs inoculated with 10^2^ HAD_50_ ASFV-G-ΔA104R had an 80% survival rate over the 28-day observation period; however, all pigs died after a lethal challenge with 10^2^ HAD_50_ parental virus (89). A151R is a nonstructural protein, pigs inoculated with 10^2^ HAD_50_ ASFV-G-ΔA151R had 80% survival during the 21-day observation period and 75% survival after the lethal challenge with the 10^2^ HAD_50_ parental virus (91). K196R is similar in sequence to the bovine pox virus thymidine kinase gene, also known as the TK gene, which encodes a viral enzyme involved in deoxyribonucleoside triphosphate synthesis (122). Because recombinant ASFV with the deleted TK gene cannot replicate in porcine macrophages, the TK gene-deleted ASFV was purified using Vero cells (123). Pigs inoculated with 10^4^ TCID_50_ or 10^6^ TCID_50_ ASFV-G/V-ΔTK survived all 21 days of the observation period and all died after receiving a lethal challenge with 10^3^ HAD_50_ ASFV-G. Some genes have a weak effect on virulence, such as C84L (98), E111R (104), L7L (115), MGF 110–9L (129), MGF300-4L (131), and MGF 360–9L (133). Deletion of other genes in the virus does not affect virulence, as seen in A224L (92), A859L (95), B125R (70), C962R (99), E66L (103), E165R (105), E184L (66), EP296R (110), I8L(L8L) (114, 115), L9R (115), L10L (115), I329L (37), K145L (75), KP177R (121), L83L (126), MGF100-1R (127), MGF110-1L (128), MGF110-5L-6L (74), MGF360-1L (132), MGF360-16R (135), O174L (137), QP509L (138), and X69R (139). However, it is still important to note that the effect of these genes on virulence may be strain-specific. These results must be verified through additional experiments.

Notably, deletion of the same gene in different strains may lead to different virulence attenuation effects (96, 97, 119, 120). For example, A238L, B119L, DP71L, DP96R, E153R, EP402R, I267L, L11L, and MGF 360–18R (DP148R). The EP402R gene encodes CD2v (8DR), a protein with significant homology to cell adhesion factors (71). CD2v is present in the outer envelope of viral particles and is responsible for binding to red blood cells (108). Once ASFV is deficient in or disrupts the CD2v protein, the virus exhibits non-HAD properties, reducing viremia *in vivo* (107). The CD2v gene affects virulence in BA71, ASFV-Kenya-IX-1033, and ASFV HLJ/18 but has no effect on virulence in the Malawi Lil-20/1 strains (107, 108). The L11L gene has no effect on the virulence of the Malawi Lil-20/1 strain but attenuates the virulence of ASFV SY18 (115, 124). Deletion of the DP148R gene in Benin 97/1 (genotype I) reduced the virulence of the strain and provided 100% protection against the parental strain (86). However, the same deletion of DP148R in ASFV Georgia 2007/1 (genotype II) and ASFV HLJ18 (genotype II) did not diminish virulence in pigs (56, 75). Deletion of the 9Gl gene in the highly virulent ASFV isolates Malawi Lil-20/1 and Pretoriuskop/96/4 resulted in a complete reduction in the virulence of the virus in pigs (16, 97). However, deletion of the 9Gl gene in the highly virulent strain ASFV Georgia 2007/1 had less effect on virulence and was dose-dependent in pigs (96). Moreover, animals inoculated with 10^2^ HAD_50_ ASFV-G-Δ9GL received only partial protection after 21 days of infection, but complete protection at 28 days. The UK gene completely attenuates the E70 strain but does not affect the virulence of ASFV Georgia 2010 (68, 102). The NL gene completely attenuated the E70 strain and affected the virulence of ASFV Georgia 2010 but was not a virulence-essential gene in Malawi Lil-20/1 and Pretoriuskop/96/4 (101). I267L has a differential effect on virulence, even when deleted in the same genotype as the ASFV (119, 120).

## DISCUSSION

African swine fever has been spreading rapidly worldwide since 2007, and because of the lack of a vaccine, the only method to stop the spread of the disease is by culling infected animals. However, this strategy is inefficient and extremely labor-intensive; therefore, an effective vaccine is urgently needed to solve this problem (1). The complexity of the ASFV genome is a major factor contributing to the delays in vaccine development. A commercially available vaccine or drug for ASFV remains elusive, when an ASF outbreak is detected in an area, the mortality rate of pigs is close to 100%, and all pigs in the area are slaughtered to control the virus. Traditional inactivated vaccines for ASF do not provide protection even when different adjuvants and inactivation methods are used (13). Live attenuated viruses offer an appropriate level of protection, with good immunogenicity and long antibody duration; however, their side effects must be considered (21, 22). Subunit vaccines are safe and have negligible side effects in pigs; although protective antigen combinations have not been identified, using this vaccine development strategy is difficult (142). However, the use of LAVs remains controversial because of their inherent infectiousness and the safety-related factors required to make LAVs usable as vaccines.

In 2022, the first ASF vaccine was marketed in Vietnam. ASFV-G-ΔI177L induced protection against the field strain of ASFV in Vietnam (118). ASFV-G-ΔI177L was immunized at different doses intramuscularly to pigs of local breeds in Vietnam and all pigs survived during the immunization period except for three deaths of pigs immunized with 10 HAD_50_

Pigs of local breeds in Vietnam were immunized intramuscularly with different doses of ASFV-G-ΔI177L (10 HAD_50_, 10^2.0^ HAD_50_, 10^3.0^ HAD_50_, or 10^4.0^ HAD_50_), and all pigs survived both the immunization period and the challenge period, except for three pigs immunized with 10 HAD_50_, which died during the challenge (118). However, ASFV-G-ΔI177L, which became available in June 2022, was suspended in August of the same year owing to pig deaths following vaccination with this vaccine. Starting in February 2023, Vietnam plans to use ASFV-G-ΔMGF, the second ASF vaccine, for widespread nationwide distribution. Harbin Veterinary Research Institute developed HLJ/18–6GD with the same gene deleted from ASFV-G-ΔMGF, and HLJ/18–6GD is also fully attenuated and resistant to lethal attack by parental viruses (56). Both vaccines have been approved for commercial trials in Vietnam. However, more questions need to be addressed to promote LAVs to the world, such as cross-protection ability, duration of protection, whether virulence will return, horizontal or vertical transmission ability, safety for pregnant sows, and more research data are needed to prove the safety of the vaccine. In China, questions persist regarding the use of LAVs. China is a major pig breeding country and numerous pigs have been rapidly infected since the emergence of ASF in 2018. The prolonged epidemic has led to the rapid evolution of the virus and the discovery of multiple natural mutants, thus making national eradication of the disease difficult (2, 11, 28, 32). The emergence of low virulence natural variant genotype II strains, genotype I strains, and genotype I and genotype II recombinant ASFVs has made the spread of ASF more insidious, and the early diagnosis of ASF more difficult, and has brought new challenges to the control of ASFV. Moreover, weakly virulent ASFV strains have safety concerns related to virulence reversal. Even the use of weak strains can easily lead to the same catastrophic consequences as those observed in the Spanish field trials. Hence, China must carefully consider the use of LAVs.

Although LAVs have the advantage of complete protection against virulent virus challenges, the following issues must be considered for vaccine candidates. First, ASFV can only grow stably in porcine primary macrophages, and although some studies have found that ASFVs can replicate effectively in PIPEC or ZMAC-4, further studies are needed (43, 53). Long-term experiments to observe the production stability of cell lines or establish other stable cell lines are required to meet vaccine production standards. Second, animals vaccinated with LAVs may experience a transient and mild temperature rise, viremia, and viral excretion through oral, nasal, or anal routes. The environmental safety of viral excretion in pigs still needs to be considered, and the safety of other pathogenic microorganisms and vaccines during fever in LAV-vaccinated animals has not yet been verified. Third, the impact of the continued spread of attenuated ASFV strains on environmental safety cannot be predicted. Given the possibility of homologous recombination among different attenuated strains to produce virulent viruses and result in epidemics, this strategy should be approached with caution. Fourth, all LAVs are still in the laboratory phase and confer protection against homologous challenge 1–3 months after LAV injection. However, ASFV can be classified into 24 genotypes based on the B646L gene, and more research is needed on cross-protective efficacy against heterologous types and the duration of LAV effectiveness.

## References

[1] Bosch-Camós L, López E, Rodriguez F. 2020. African swine fever vaccines: a promising work still in progress. Porc Health Manag 6:17. doi:10.1186/s40813-020-00154-2 PMC7329361 32626597

[2] Zhao D, Sun E, Huang L, Ding L, Zhu Y, Zhang J, Shen D, Zhang X, Zhang Z, Ren T, Wang W, Li F, He X, Bu Z. 2023. Highly lethal genotype I and II recombinant African swine fever viruses detected in pigs. Nat Commun 14:3096. doi:10.1038/s41467-023-38868-w 37248233 PMC10226439

[3] Zhu JJ. 2022. African swine fever vaccinology: the biological challenges from immunological perspectives. Viruses 14:2021. doi:10.3390/v14092021 36146827 PMC9505361

[4] Bishop RP, Fleischauer C, de Villiers EP, Okoth EA, Arias M, Gallardo C, Upton C. 2015. Comparative analysis of the complete genome sequences of Kenyan African swine fever virus isolates within p72 genotypes IX and X. Virus Genes 50:303–309. doi:10.1007/s11262-014-1156-7 25645905

[5] Chapman DAG, Tcherepanov V, Upton C, Dixon LK. 2008. Comparison of the genome sequences of non-pathogenic and pathogenic African swine fever virus isolates. J Gen Virol 89:397–408. doi:10.1099/vir.0.83343-0 18198370

[6] Wang T, Luo R, Sun Y, Qiu H-J. 2021. Current efforts towards safe and effective live attenuated vaccines against African swine fever: challenges and prospects. Infect Dis Poverty 10:137. doi:10.1186/s40249-021-00920-6 34949228 PMC8702042

[7] Wang G, Xie M, Wu W, Chen Z. 2021. Structures and functional diversities of ASFV proteins. Viruses 13:2124. doi:10.3390/v13112124 34834930 PMC8619059

[8] Blasco R, Agüero M, Almendral JM, Viñuela E. 1989. Variable and constant regions in African swine fever virus DNA. Virology (Auckl) 168:330–338. doi:10.1016/0042-6822(89)90273-0 2464873

[9] Hakizimana JN, Nyabongo L, Ntirandekura JB, Yona C, Ntakirutimana D, Kamana O, Nauwynck H, Misinzo G. 2020. Genetic analysis of African swine fever virus from the 2018 outbreak in South-Eastern Burundi. Front Vet Sci 7:578474. doi:10.3389/fvets.2020.578474 33251264 PMC7674587

[10] Bastos ADS, Penrith M-L, Crucière C, Edrich JL, Hutchings G, Roger F, Couacy-Hymann E, R Thomson G. 2003. Genotyping field strains of African swine fever virus by partial p72 gene characterisation. Arch Virol 148:693–706. doi:10.1007/s00705-002-0946-8 12664294

[11] Zhou X, Li N, Luo Y, Liu Y, Miao F, Chen T, Zhang S, Cao P, Li X, Tian K, Qiu H-J, Hu R. 2018. Emergence of African swine fever in China, 2018. Transbound Emerg Dis 65:1482–1484. doi:10.1111/tbed.12989 30102848

[12] Rowlands RJ, Michaud V, Heath L, Hutchings G, Oura C, Vosloo W, Dwarka R, Onashvili T, Albina E, Dixon LK. 2008. African swine fever virus isolate, Georgia, 2007. Emerg Infect Dis 14:1870–1874. doi:10.3201/eid1412.080591 19046509 PMC2634662

[13] Blome S, Gabriel C, Beer M. 2014. Modern adjuvants do not enhance the efficacy of an inactivated African swine fever virus vaccine preparation. Vaccine (Auckl) 32:3879–3882. doi:10.1016/j.vaccine.2014.05.051 24877766

[14] Cadenas-Fernández E, Sánchez-Vizcaíno JM, van den Born E, Kosowska A, van Kilsdonk E, Fernández-Pacheco P, Gallardo C, Arias M, Barasona JA. 2021. High doses of inactivated African swine fever virus are safe, but do not confer protection against a virulent challenge. Vaccines (Basel) 9:242. doi:10.3390/vaccines9030242 33802021 PMC7999564

[15] Urbano AC, Ferreira F. 2022. African swine fever control and prevention: an update on vaccine development. Emerg Microbes Infect 11:2021–2033. doi:10.1080/22221751.2022.2108342 35912875 PMC9423837

[16] Neilan JG, Zsak L, Lu Z, Burrage TG, Kutish GF, Rock DL. 2004. Neutralizing antibodies to African swine fever virus proteins p30, p54, and p72 are not sufficient for antibody-mediated protection. Virology (Auckl) 319:337–342. doi:10.1016/j.virol.2003.11.011 14980493

[17] Lokhandwala S, Petrovan V, Popescu L, Sangewar N, Elijah C, Stoian A, Olcha M, Ennen L, Bray J, Bishop RP, Waghela SD, Sheahan M, Rowland RRR, Mwangi W. 2019. Adenovirus-vectored African swine fever virus antigen cocktails are immunogenic but not protective against intranasal challenge with Georgia 2007/1 isolate. Vet Microbiol 235:10–20. doi:10.1016/j.vetmic.2019.06.006 31282366

[18] Lokhandwala S, Waghela SD, Bray J, Sangewar N, Charendoff C, Martin CL, Hassan WS, Koynarski T, Gabbert L, Burrage TG, Brake D, Neilan J, Mwangi W. 2017. Adenovirus-vectored novel African swine fever virus antigens elicit robust immune responses in swine. PLoS One 12:e0177007. doi:10.1371/journal.pone.0177007 28481911 PMC5421782

[19] Argilaguet JM, Pérez-Martín E, López S, Goethe M, Escribano JM, Giesow K, Keil GM, Rodríguez F. 2013. BacMam immunization partially protects pigs against sublethal challenge with African swine fever virus. Antiviral Res 98:61–65. doi:10.1016/j.antiviral.2013.02.005 23428670

[20] Monteagudo PL, Lacasta A, López E, Bosch L, Collado J, Pina-Pedrero S, Correa-Fiz F, Accensi F, Navas MJ, Vidal E, Bustos MJ, Rodríguez JM, Gallei A, Nikolin V, Salas ML, Rodríguez F. 2017. BA71ΔCD2: a new recombinant live attenuated African swine fever virus with cross-protective capabilities. J Virol 91:e01058-17. doi:10.1128/JVI.01058-17 28814514 PMC5640839

[21] Zhang Y, Ke J, Zhang J, Yang J, Yue H, Zhou X, Qi Y, Zhu R, Miao F, Li Q, Zhang F, Wang Y, Han X, Mi L, Yang J, Zhang S, Chen T, Hu R. 2021. African swine fever virus bearing an I226R gene deletion elicits robust immunity in pigs to African swine fever. J Virol 95:e0119921. doi:10.1128/JVI.01199-21 34495696 PMC8577359

[22] Borca MV, Ramirez-Medina E, Silva E, Vuono E, Rai A, Pruitt S, Holinka LG, Velazquez-Salinas L, Zhu J, Gladue DP. 2020. Development of a highly effective African swine fever virus vaccine by deletion of the I177L gene results in sterile immunity against the current epidemic Eurasia strain. J Virol 94:e02017-19. doi:10.1128/JVI.02017-19 31969432 PMC7081903

[23] Arias M, de la Torre A, Dixon L, Gallardo C, Jori F, Laddomada A, Martins C, Parkhouse RM, Revilla Y, Rodriguez F-M, Sanchez-Vizcaino. 2017. Approaches and perspectives for development of African swine fever virus vaccines. Vaccines (Basel) 5:35. doi:10.3390/vaccines5040035 28991171 PMC5748602

[24] King K, Chapman D, Argilaguet JM, Fishbourne E, Hutet E, Cariolet R, Hutchings G, Oura CAL, Netherton CL, Moffat K, Taylor G, Le Potier M-F, Dixon LK, Takamatsu H-H. 2011. Protection of European domestic pigs from virulent African isolates of African swine fever virus by experimental immunisation. Vaccine (Auckl) 29:4593–4600. doi:10.1016/j.vaccine.2011.04.052 PMC3120964 21549789

[25] Sánchez-Cordón PJ, Chapman D, Jabbar T, Reis AL, Goatley L, Netherton CL, Taylor G, Montoya M, Dixon L. 2017. Different routes and doses influence protection in pigs immunised with the naturally attenuated African swine fever virus isolate OURT88/3. Antiviral Res 138:1–8. doi:10.1016/j.antiviral.2016.11.021 27908827 PMC5245086

[26] Leitão A, Cartaxeiro C, Coelho R, Cruz B, Parkhouse RME, Portugal FC, Vigário JD, Martins CLV. 2001. The non-haemadsorbing African swine fever virus isolate ASFV/NH/P68 provides a model for defining the protective anti-virus immune response. J Gen Virol 82:513–523. doi:10.1099/0022-1317-82-3-513 11172092

[27] Gallardo C, Sánchez EG, Pérez-Núñez D, Nogal M, de León P, Carrascosa ÁL, Nieto R, Soler A, Arias ML, Revilla Y. 2018. African swine fever virus (ASFV) protection mediated by NH/P68 and NH/P68 recombinant live-attenuated viruses. Vaccine (Auckl) 36:2694–2704. doi:10.1016/j.vaccine.2018.03.040 29609966

[28] Sun E, Huang L, Zhang X, Zhang J, Shen D, Zhang Z, Wang Z, Huo H, Wang W, Huangfu H, Wang W, Li F, Liu R, Sun J, Tian Z, Xia W, Guan Y, He X, Zhu Y, Zhao D, Bu Z. 2021. Genotype I African swine fever viruses emerged in domestic pigs in China and caused chronic infection. Emerg Microbes Infect 10:2183–2193. doi:10.1080/22221751.2021.1999779 34709128 PMC8635679

[29] Gallardo C, Soler A, Rodze I, Nieto R, Cano-Gómez C, Fernandez-Pinero J, Arias M. 2019. Attenuated and non-haemadsorbing (non-HAD) genotype II African swine fever virus (ASFV) isolated in Europe, Latvia 2017. Transbound Emerg Dis 66:1399–1404. doi:10.1111/tbed.13132 30667598

[30] Barroso-Arévalo S, Barasona JA, Cadenas-Fernández E, Sánchez-Vizcaíno JM. 2021. The role of interleukine-10 and interferon-γ as potential markers of the evolution of African swine fever virus infection in wild boar. Pathogens 10:757. doi:10.3390/pathogens10060757 34203976 PMC8232672

[31] Tamás V, Righi C, Mészáros I, D’Errico F, Olasz F, Casciari C, Zádori Z, Magyar T, Petrini S, Feliziani F. 2023. Involvement of the MGF 110-11L gene in the African swine fever replication and virulence. Vaccines (Basel) 11:846. doi:10.3390/vaccines11040846 37112759 PMC10145817

[32] Sun E, Zhang Z, Wang Z, He X, Zhang X, Wang L, Wang W, Huang L, Xi F, Huangfu H, Tsegay G, Huo H, Sun J, Tian Z, Xia W, Yu X, Li F, Liu R, Guan Y, Zhao D, Bu Z. 2021. Emergence and prevalence of naturally occurring lower virulent African swine fever viruses in domestic pigs in China in 2020. Sci China Life Sci 64:752–765. doi:10.1007/s11427-021-1904-4 33655434

[33] Ramirez-Medina E, O’Donnell V, Silva E, Espinoza N, Velazquez-Salinas L, Moran K, Daite DA, Barrette R, Faburay B, Holland R, Gladue DP, Borca MV. 2022. Experimental infection of domestic pigs with an African swine fever virus field strain isolated in 2021 from the Dominican Republic. Viruses 14:1090. doi:10.3390/v14051090 35632831 PMC9145207

[34] Vigário JD, Terrinha AM, Moura Nunes JF. 1974. Antigenic relationships among strains of African swine fecre virus. Arch Gesamte Virusforsch 45:272–277. doi:10.1007/BF01249690 4138464

[35] Boinas FS, Hutchings GH, Dixon LK, Wilkinson PJ. 2004. Characterization of pathogenic and non-pathogenic African swine fever virus isolates from Ornithodoros erraticus inhabiting pig premises in Portugal. J Gen Virol 85:2177–2187. doi:10.1099/vir.0.80058-0 15269356

[36] Abrams CC, Goatley L, Fishbourne E, Chapman D, Cooke L, Oura CA, Netherton CL, Takamatsu H-H, Dixon LK. 2013. Deletion of virulence associated genes from attenuated African swine fever virus isolate OUR T88/3 decreases its ability to protect against challenge with virulent virus. Virol (Auckl) 443:99–105. doi:10.1016/j.virol.2013.04.028 PMC3709090 23725691

[37] Reis AL, Goatley LC, Jabbar T, Lopez E, Rathakrishnan A, Dixon LK. 2020. Deletion of the gene for the type I interferon inhibitor I329L from the attenuated African swine fever virus OURT88/3 strain reduces protection induced in pigs. Vaccines (Basel) 8:262. doi:10.3390/vaccines8020262 32486154 PMC7349983

[38] Meloni D, Franzoni G, Oggiano A. 2022. Cell lines for the development of African swine fever virus vaccine candidates: an update. Vaccines (Basel) 10:707. doi:10.3390/vaccines10050707 35632463 PMC9144233

[39] de León P, Bustos MJ, Carrascosa AL. 2013. Laboratory methods to study African swine fever virus. Virus Res 173:168–179. doi:10.1016/j.virusres.2012.09.013 23041357

[40] Krug PW, Holinka LG, O’Donnell V, Reese B, Sanford B, Fernandez-Sainz I, Gladue DP, Arzt J, Rodriguez L, Risatti GR, Borca MV. 2015. The progressive adaptation of a georgian isolate of African swine fever virus to vero cells leads to a gradual attenuation of virulence in swine corresponding to major modifications of the viral genome. J Virol 89:2324–2332. doi:10.1128/JVI.03250-14 25505073 PMC4338881

[41] Carrascosa AL, Bustos MJ, de Leon P. 2011. Methods for growing and titrating African swine fever virus: field and laboratory samples. Curr Protoc Cell Biol Chapter 26:26. doi:10.1002/0471143030.cb2614s53 22161547

[42] Wang T, Wang L, Han Y, Pan L, Yang J, Sun M, Zhou P, Sun Y, Bi Y, Qiu H-J. 2021. Adaptation of African swine fever virus to HEK293T cells. Transbound Emerg Dis 68:2853–2866. doi:10.1111/tbed.14242 34314096

[43] Portugal R, Goatley LC, Husmann R, Zuckermann FA, Dixon LK. 2020. A porcine macrophage cell line that supports high levels of replication of OURT88/3, an attenuated strain of African swine fever virus. Emerg Microbes Infect 9:1245–1253. doi:10.1080/22221751.2020.1772675 32515659 PMC7448849

[44] Borca MV, Rai A, Espinoza N, Ramirez-Medina E, Spinard E, Velazquez-Salinas L, Valladares A, Silva E, Burton L, Meyers A, Gay CG, Gladue DP. 2023. African swine fever vaccine candidate ASFV-G-ΔI177L produced in the swine macrophage-derived cell line IPKM remains genetically stable and protective against homologous virulent challenge. Viruses 15:2064. doi:10.3390/v15102064 37896841 PMC10612016

[45] Enjuanes L, Carrascosa AL, Moreno MA, Viñuela E. 1976. Titration of African swine fever (ASF) virus. J Gen Virol 32:471–477. doi:10.1099/0022-1317-32-3-471 823294

[46] Rodríguez JM, Moreno LT, Alejo A, Lacasta A, Rodríguez F, Salas ML. 2015. Genome sequence of African swine fever virus BA71, the virulent parental strain of the nonpathogenic and tissue-culture adapted BA71V. PLoS One 10:e0142889. doi:10.1371/journal.pone.0142889 26618713 PMC4664411

[47] Koltsova G, Koltsov A, Krutko S, Kholod N, Tulman ER, Kolbasov D. 2021. Growth kinetics and protective efficacy of attenuated ASFV strain congo with deletion of the EP402 gene. Viruses 13:1259. doi:10.3390/v13071259 34203302 PMC8309992

[48] Tabarés E, Olivares I, Santurde G, Garcia MJ, Martin E, Carnero ME. 1987. African swine fever virus DNA: deletions and additions during adaptation to growth in monkey kidney cells. Arch Virol 97:333–346. doi:10.1007/BF01314431 2827611

[49] Zsak L, Onisk DV, Afonso CL, Rock DL. 1993. Virulent African swine fever virus isolates are neutralized by swine immune serum and by monoclonal antibodies recognizing a 72-kDa viral protein. Virology (Auckl) 196:596–602. doi:10.1006/viro.1993.1515 7690502

[50] Truong QL, Wang L, Nguyen TA, Nguyen HT, Tran SD, Vu AT, Le AD, Nguyen VG, Hoang PT, Nguyen YT, Le TL, Van TN, Huynh TML, Lai HTL, Madera R, Li Y, Shi J, Nguyen LT. 2023. A cell-adapted live-attenuated vaccine candidate protects pigs against the homologous strain VNUA-ASFV-05L1, a representative strain of the contemporary pandemic African swine fever virus. Viruses 15:2089. doi:10.3390/v15102089 37896866 PMC10612049

[51] Petrini S, Righi C, Mészáros I, D’Errico F, Tamás V, Pela M, Olasz F, Gallardo C, Fernandez-Pinero J, Göltl E, Magyar T, Feliziani F, Zádori Z. 2023. The production of recombinant African swine fever virus Lv17/WB/Rie1 strains and their in vitro and in vivo characterizations. Vaccines (Basel) 11:11860. doi:10.3390/vaccines11121860 PMC10748256 38140263

[52] Bourry O, Hutet E, Le Dimna M, Lucas P, Blanchard Y, Chastagner A, Paboeuf F, Le Potier M-F. 2022. Oronasal or intramuscular immunization with a thermo-attenuated ASFV strain provides full clinical protection against Georgia 2007/1 challenge. Viruses 14:2777. doi:10.3390/v14122777 36560781 PMC9784117

[53] Borca MV, Rai A, Ramirez-Medina E, Silva E, Velazquez-Salinas L, Vuono E, Pruitt S, Espinoza N, Gladue DP. 2021. A cell culture-adapted vaccine virus against the current African swine fever virus pandemic strain. J Virol 95:e0012321. doi:10.1128/JVI.00123-21 33952643 PMC8315737

[54] Hemmink JD, Abkallo HM, Henson SP, Khazalwa EM, Oduor B, Lacasta A, Okoth E, Riitho V, Fuchs W, Bishop RP, Steinaa L. 2022. The African swine fever isolate ASFV-Kenya-IX-1033 is highly virulent and stable after propagation in the wild boar cell line WSL. Viruses 14:1912. doi:10.3390/v14091912 36146718 PMC9505471

[55] Li D, Wu P, Liu H, Feng T, Yang W, Ru Y, Li P, Qi X, Shi Z, Zheng H. 2022. A QP509L/QP383R-deleted African swine fever virus is highly attenuated in swine but does not confer protection against parental virus challenge. J Virol 96:e0150021. doi:10.1128/JVI.01500-21 34613824 PMC8754219

[56] Chen W, Zhao D, He X, Liu R, Wang Z, Zhang X, Li F, Shan D, Chen H, Zhang J, Wang L, Wen Z, Wang X, Guan Y, Liu J, Bu Z. 2020. A seven-gene-deleted African swine fever virus is safe and effective as a live attenuated vaccine in pigs. Sci China Life Sci 63:623–634. doi:10.1007/s11427-020-1657-9 32124180 PMC7223596

[57] O’Donnell V, Holinka LG, Sanford B, Krug PW, Carlson J, Pacheco JM, Reese B, Risatti GR, Gladue DP, Borca MV. 2016. African swine fever virus Georgia isolate harboring deletions of 9GL and MGF360/505 genes is highly attenuated in swine but does not confer protection against parental virus challenge. Virus Res 221:8–14. doi:10.1016/j.virusres.2016.05.014 27182007

[58] Sánchez-Cordón PJ, Jabbar T, Berrezaie M, Chapman D, Reis A, Sastre P, Rueda P, Goatley L, Dixon LK. 2018. Evaluation of protection induced by immunisation of domestic pigs with deletion mutant African swine fever virus BeninΔMGF by different doses and routes. Vaccine (Auckl) 36:707–715. doi:10.1016/j.vaccine.2017.12.030 PMC5783716 29254837

[59] Pérez-Núñez D, Sunwoo S-Y, García-Belmonte R, Kim C, Vigara-Astillero G, Riera E, Kim D-M, Jeong J, Tark D, Ko Y-S, You Y-K, Revilla Y. 2022. Recombinant African swine fever virus Arm/07/CBM/c2 lacking CD2v and A238L is attenuated and protects pigs against virulent Korean Paju strain. Vaccines (Basel) 10:1992. doi:10.3390/vaccines10121992 36560402 PMC9784410

[60] Teklue T, Wang T, Luo Y, Hu R, Sun Y, Qiu H-J. 2020. Generation and evaluation of an African swine fever virus mutant with deletion of the CD2v and UK genes. Vaccines 8:763. doi:10.3390/vaccines8040763 33327488 PMC7768475

[61] Borca MV, Ramirez-Medina E, Espinoza N, Rai A, Spinard E, Velazquez-Salinas L, Valladares A, Silva E, Burton L, Meyers A, Clark J, Wu P, Gay CG, Gladue DP. 2024. Deletion of the EP402R gene from the genome of African swine fever vaccine strain ASFV-G-∆I177L provides the potential capability of differentiating between infected and vaccinated animals. Viruses 16:376. doi:10.3390/v16030376 38543742 PMC10974803

[62] Li J, Song J, Zhou S, Li S, Liu J, Li T, Zhang Z, Zhang X, He X, Chen W, Zheng J, Zhao D, Bu Z, Huang L, Weng C. 2023. Development of a new effective African swine fever virus vaccine candidate by deletion of the H240R and MGF505-7R genes results in protective immunity against the Eurasia strain. J Virol 97:e0070423. doi:10.1128/jvi.00704-23 37768081 PMC10617561

[63] Li D, Ren J, Zhu G, Wu P, Yang W, Ru Y, Feng T, Liu H, Zhang J, Peng J, Tian H, Liu X, Zheng H. 2023. Deletions of MGF110-9L and MGF360-9L from African swine fever virus are highly attenuated in swine and confer protection against homologous challenge. J Biol Chem 299:104767. doi:10.1016/j.jbc.2023.104767 37142221 PMC10236468

[64] Deutschmann P, Carrau T, Sehl-Ewert J, Forth JH, Viaplana E, Mancera JC, Urniza A, Beer M, Blome S. 2022. Taking a promising vaccine candidate further: efficacy of ASFV-G-ΔMGF after intramuscular vaccination of domestic pigs and oral vaccination of wild boar. Pathogens 11:996. doi:10.3390/pathogens11090996 36145428 PMC9504512

[65] Qi X, Feng T, Ma Z, Zheng L, Liu H, Shi Z, Shen C, Li P, Wu P, Ru Y, Li D, Zhu Z, Tian H, Wu S, Zheng H. 2023. Deletion of DP148R, DP71L, and DP96R attenuates African swine fever virus, and the mutant strain confers complete protection against homologous challenges in pigs. J Virol 97:e0024723. doi:10.1128/jvi.00247-23 37017515 PMC10134827

[66] Ramirez-Medina E, Vuono E, Rai A, Pruitt S, Espinoza N, Velazquez-Salinas L, Pina-Pedrero S, Zhu J, Rodriguez F, Borca MV, Gladue DP. 2022. Deletion of E184L, a putative DIVA target from the pandemic strain of African swine fever virus, produces a reduction in virulence and protection against virulent challenge. J Virol 96:e0141921. doi:10.1128/JVI.01419-21 34668772 PMC8754217

[67] Gladue DP, O’Donnell V, Ramirez-Medina E, Rai A, Pruitt S, Vuono EA, Silva E, Velazquez-Salinas L, Borca MV. 2020. Deletion of CD2-Like (CD2v) and C-type lectin-like (EP153R) genes from African swine fever virus Georgia-∆9GL abrogates its effectiveness as an experimental vaccine. Viruses 12:1185. doi:10.3390/v12101185 33092057 PMC7590024

[68] Ramirez-Medina E, Vuono E, O’Donnell V, Holinka LG, Silva E, Rai A, Pruitt S, Carrillo C, Gladue DP, Borca MV. 2019. Differential effect of the deletion of African swine fever virus virulence-associated genes in the induction of attenuation of the highly virulent Georgia strain. Viruses 11:599. doi:10.3390/v11070599 31269702 PMC6669436

[69] Reis AL, Rathakrishnan A, Goulding LV, Barber C, Goatley LC, Dixon LK. 2023. Deletion of the gene for the African swine fever virus BCL-2 family member A179L increases virus uptake and apoptosis but decreases virus spread in macrophages and reduces virulence in pigs. J Virol 97:e0110623. doi:10.1128/jvi.01106-23 37796125 PMC10617521

[70] Zhu R, Wang Y, Zhang H, Yang J, Fan J, Zhang Y, Wang Y, Li Q, Zhou X, Yue H, Qi Y, Wang S, Chen T, Zhang S, Hu R. 2024. Deletion of the B125R gene in the African swine fever virus SY18 strain leads to an A104R frameshift mutation slightly attenuating virulence in domestic pigs. Virus Res 343:199343. doi:10.1016/j.virusres.2024.199343 38423214 PMC10982076

[71] Abkallo HM, Hemmink JD, Oduor B, Khazalwa EM, Svitek N, Assad-Garcia N, Khayumbi J, Fuchs W, Vashee S, Steinaa L. 2022. Co-deletion of A238L and EP402R genes from a genotype IX African swine fever virus results in partial attenuation and protection in swine. Viruses 14:2024. doi:10.3390/v14092024 36146830 PMC9501025

[72] O’Donnell V, Risatti GR, Holinka LG, Krug PW, Carlson J, Velazquez-Salinas L, Azzinaro PA, Gladue DP, Borca MV. 2017. Simultaneous deletion of the 9GL and UK genes from the African swine fever virus Georgia 2007 isolate offers increased safety and protection against homologous challenge. J Virol 91:e01760-16. doi:10.1128/JVI.01760-16 27795430 PMC5165186

[73] Lopez E, Bosch-Camós L, Ramirez-Medina E, Vuono E, Navas MJ, Muñoz M, Accensi F, Zhang J, Alonso U, Argilaguet J, Salas ML, Anachkov N, Gladue DP, Borca MV, Pina-Pedrero S, Rodriguez F. 2021. Deletion mutants of the attenuated recombinant ASF virus, BA71ΔCD2, show decreased vaccine efficacy. Viruses 13:1678. doi:10.3390/v13091678 34578263 PMC8473413

[74] Ramirez-Medina E, Vuono E, Silva E, Rai A, Valladares A, Pruitt S, Espinoza N, Velazquez-Salinas L, Borca MV, Gladue DP. 2022. Evaluation of the deletion of MGF110-5L-6L on swine virulence from the pandemic strain of African swine fever virus and use as a DIVA marker in vaccine candidate ASFV-G-ΔI177L. J Virol 96:e0059722. doi:10.1128/jvi.00597-22 35862688 PMC9327674

[75] Rathakrishnan A, Reis AL, Goatley LC, Moffat K, Dixon LK. 2021. Deletion of the K145R and DP148R genes from the virulent ASFV Georgia 2007/1 isolate delays the onset, but does not reduce severity, of clinical signs in infected pigs. Viruses 13:1473. doi:10.3390/v13081473 34452339 PMC8402900

[76] Ding M, Dang W, Liu H, Zhang K, Xu F, Tian H, Huang H, Shi Z, Sunkang Y, Qin X, Zhang Y, Zheng H. 2022. Sequential deletions of interferon inhibitors MGF110-9L and MGF505-7R result in sterile immunity against the Eurasia strain of Africa swine fever. J Virol 96:e0119222. doi:10.1128/jvi.01192-22 36197109 PMC9599437

[77] Ding M, Dang W, Liu H, Xu F, Huang H, Sunkang Y, Li T, Pei J, Liu X, Zhang Y, Zheng H. 2022. Combinational deletions of MGF360-9L and MGF505-7R attenuated highly virulent African swine fever virus and conferred protection against homologous challenge. J Virol 96:e0032922. doi:10.1128/jvi.00329-22 35867564 PMC9327683

[78] Zhang J, Zhang Y, Chen T, Yang J, Yue H, Wang L, Zhou X, Qi Y, Han X, Ke J, Wang S, Yang J, Miao F, Zhang S, Zhang F, Wang Y, Li M, Hu R. 2021. Deletion of the L7L-L11L genes attenuates ASFV and induces protection against homologous challenge. Viruses 13:255. doi:10.3390/v13020255 33567491 PMC7915138

[79] Rathakrishnan A, Connell S, Petrovan V, Moffat K, Goatley LC, Jabbar T, Sánchez-Cordón PJ, Reis AL, Dixon LK. 2022. Differential effect of deleting members of African swine fever virus multigene families 360 and 505 from the genotype II Georgia 2007/1 isolate on virus replication, virulence, and induction of protection. J Virol 96:e0189921. doi:10.1128/jvi.01899-21 35044212 PMC8941908

[80] Xie Z, Liu Y, Di D, Liu J, Gong L, Chen Z, Li Y, Yu W, Lv L, Zhong Q, Song Y, Liao X, Song Q, Wang H, Chen H. 2022. Protection evaluation of a five-gene-deleted African swine fever virus vaccine candidate against homologous challenge. Front Microbiol 13:902932. doi:10.3389/fmicb.2022.902932 35966648 PMC9374035

[81] O’Donnell V, Holinka LG, Gladue DP, Sanford B, Krug PW, Lu X, Arzt J, Reese B, Carrillo C, Risatti GR, Borca MV. 2015. African swine fever virus Georgia isolate harboring deletions of MGF360 and MGF505 genes is attenuated in swine and confers protection against challenge with virulent parental virus. J Virol 89:6048–6056. doi:10.1128/JVI.00554-15 25810553 PMC4442422

[82] Neilan JG, Zsak L, Lu Z, Kutish GF, Afonso CL, Rock DL. 2002. Novel swine virulence determinant in the left variable region of the African swine fever virus genome. J Virol 76:3095–3104. doi:10.1128/jvi.76.7.3095-3104.2002 11884534 PMC136047

[83] Kitamura T, Masujin K, Yamazoe R, Kameyama K-I, Watanabe M, Ikezawa M, Yamada M, Kokuho T. 2023. A spontaneously occurring African swine fever virus with 11 gene deletions partially protects pigs challenged with the parental strain. Viruses 15:311. doi:10.3390/v15020311 36851524 PMC9966947

[84] Lopez E, van Heerden J, Bosch-Camós L, Accensi F, Navas MJ, López-Monteagudo P, Argilaguet J, Gallardo C, Pina-Pedrero S, Salas ML, Salt J, Rodriguez F. 2020. Live attenuated African swine fever viruses as ideal tools to dissect the mechanisms involved in cross-protection. Viruses 12:1474. doi:10.3390/v12121474 33371460 PMC7767464

[85] Titov I, Burmakina G, Morgunov Y, Morgunov S, Koltsov A, Malogolovkin A, Kolbasov D. 2017. Virulent strain of African swine fever virus eclipses its attenuated derivative after challenge. Arch Virol 162:3081–3088. doi:10.1007/s00705-017-3471-5 28691128

[86] Reis AL, Goatley LC, Jabbar T, Sanchez-Cordon PJ, Netherton CL, Chapman DAG, Dixon LK. 2017. Deletion of the African swine fever virus gene DP148R does not reduce virus replication in culture but reduces virus virulence in pigs and induces high levels of protection against challenge. J Virol 91:e01428-17. doi:10.1128/JVI.01428-17 PMC5709585 28978700

[87] Petrovan V, Rathakrishnan A, Islam M, Goatley LC, Moffat K, Sanchez-Cordon PJ, Reis AL, Dixon LK. 2022. Role of African swine fever virus proteins EP153R and EP402R in reducing viral persistence in blood and virulence in pigs infected with BeninΔDP148R. J Virol 96:e0134021. doi:10.1128/JVI.01340-21 34643433 PMC8754224

[88] Liu Y, Xie Z, Li Y, Song Y, Di D, Liu J, Gong L, Chen Z, Wu J, Ye Z, Liu J, Yu W, Lv L, Zhong Q, Tian C, Song Q, Wang H, Chen H. 2023. Evaluation of an I177L gene-based five-gene-deleted African swine fever virus as a live attenuated vaccine in pigs. Emerg Microbes Infect 12:2148560. doi:10.1080/22221751.2022.2148560 36378022 PMC9769145

[89] Ramirez-Medina E, Vuono EA, Pruitt S, Rai A, Espinoza N, Valladares A, Silva E, Velazquez-Salinas L, Borca MV, Gladue DP. 2022. Deletion of African swine fever virus histone-like protein, A104R from the Georgia isolate drastically reduces virus virulence in domestic pigs. Viruses 14:1112. doi:10.3390/v14051112 35632853 PMC9146580

[90] Gladue DP, Ramirez-Medina E, Vuono E, Silva E, Rai A, Pruitt S, Espinoza N, Velazquez-Salinas L, Borca MV. 2021. Deletion of the A137R gene from the pandemic strain of African swine fever virus attenuates the strain and offers protection against the virulent pandemic virus. J Virol 95:e0113921. doi:10.1128/JVI.01139-21 34406865 PMC8513468

[91] Ramirez-Medina E, Vuono E, Pruitt S, Rai A, Espinoza N, Valladares A, Spinard E, Silva E, Velazquez-Salinas L, Gladue DP, Borca MV. 2022. ASFV gene A151R is involved in the process of virulence in domestic swine. Viruses 14:1834. doi:10.3390/v14081834 36016456 PMC9413758

[92] Neilan JG, Lu Z, Kutish GF, Zsak L, Burrage TG, Borca MV, Carrillo C, Rock DL. 1997. A BIR motif containing gene of African swine fever virus, 4CL, is nonessential for growth in vitro and viral virulence. Virology (Auckl) 230:252–264. doi:10.1006/viro.1997.8481 9143281

[93] Salguero FJ, Gil S, Revilla Y, Gallardo C, Arias M, Martins C. 2008. Cytokine mRNA expression and pathological findings in pigs inoculated with African swine fever virus (E-70) deleted on A238L. Vet Immunol Immunopathol 124:107–119. doi:10.1016/j.vetimm.2008.02.012 18384883

[94] Neilan JG, Lu Z, Kutish GF, Zsak L, Lewis TL, Rock DL. 1997. A conserved African swine fever virus IkappaB homolog, 5EL, is nonessential for growth in vitro and virulence in domestic swine. Virol (Auckl) 235:377–385. doi:10.1006/viro.1997.8693 9281518

[95] Ramirez-Medina E, Vuono EA, Pruitt S, Rai A, Espinoza N, Velazquez-Salinas L, Gladue DP, Borca MV. 2021. Evaluation of an ASFV RNA helicase gene A859L for virus replication and swine virulence. Viruses 14:10. doi:10.3390/v14010010 35062213 PMC8777736

[96] O’Donnell V, Holinka LG, Krug PW, Gladue DP, Carlson J, Sanford B, Alfano M, Kramer E, Lu Z, Arzt J, Reese B, Carrillo C, Risatti GR, Borca MV. 2015. African swine fever virus Georgia 2007 with a deletion of virulence-associated gene 9GL (B119L), when administered at low doses, leads to virus attenuation in swine and induces an effective protection against homologous challenge. J Virol 89:8556–8566. doi:10.1128/JVI.00969-15 26063424 PMC4524225

[97] Lewis T, Zsak L, Burrage TG, Lu Z, Kutish GF, Neilan JG, Rock DL. 2000. An African swine fever virus ERV1-ALR homologue, 9GL, affects virion maturation and viral growth in macrophages and viral virulence in swine. J Virol 74:1275–1285. doi:10.1128/jvi.74.3.1275-1285.2000 10627538 PMC111462

[98] Yang J, Zhu R, Zhang Y, Zhou X, Yue H, Li Q, Ke J, Wang Y, Miao F, Chen T, Zhang F, Zhang S, Qian A, Hu R. 2024. Deleting the C84L gene from the virulent African swine fever virus SY18 does not affect Its replication in porcine primary macrophages but reduces its virulence in swine. Pathogens 13:103. doi:10.3390/pathogens13020103 38392841 PMC10891671

[99] Ramirez-Medina E, Vuono EA, Rai A, Pruitt S, Silva E, Velazquez-Salinas L, Zhu J, Borca MV, Gladue DP. 2020. The C962R ORF of African swine fever strain Georgia is non-essential and not required for virulence in swine. Viruses 12:676. doi:10.3390/v12060676 32585808 PMC7354530

[100] Zsak L, Lu Z, Kutish GF, Neilan JG, Rock DL. 1996. An African swine fever virus virulence-associated gene NL-S with similarity to the herpes simplex virus ICP34.5 gene. J Virol 70:8865–8871. doi:10.1128/JVI.70.12.8865-8871.1996 8971015 PMC190983

[101] Afonso CL, Carrillo C, Zsak L, Rock DL, Borca MV. 1998. African swine fever virus NL gene is not required for virus virulence. J Gen Virol 79:2543–2547. doi:10.1099/0022-1317-79-10-2543 9780062

[102] Zsak L, Caler E, Lu Z, Kutish GF, Neilan JG, Rock DL. 1998. A nonessential African swine fever virus gene UK is a significant virulence determinant in domestic swine. J Virol 72:1028–1035. doi:10.1128/JVI.72.2.1028-1035.1998 9444996 PMC124574

[103] Ramirez-Medina E, Vuono EA, Rai A, Espinoza N, Valladares A, Spinard E, Velazquez-Salinas L, Gladue DP, Borca MV. 2023. Evaluation of the function of ASFV gene E66L in the process of virus replication and virulence in swine. Viruses 15:566. doi:10.3390/v15020566 36851779 PMC9965554

[104] Zhou X, Fan J, Zhang Y, Yang J, Zhu R, Yue H, Qi Y, Li Q, Wang Y, Chen T, Zhang S, Hu R. 2023. Evaluation of African swine fever virus E111R gene on viral replication and porcine virulence. Viruses 15:890. doi:10.3390/v15040890 37112870 PMC10143872

[105] Vuono EA, Ramirez-Medina E, Pruitt S, Rai A, Espinoza N, Silva E, Velazquez-Salinas L, Gladue DP, Borca MV. 2022. Deletion of the ASFV dUTPase gene E165R from the genome of highly virulent African swine fever virus Georgia 2010 does not affect virus replication or virulence in domestic pigs. Viruses 14:1409. doi:10.3390/v14071409 35891389 PMC9320246

[106] Neilan JG, Borca MV, Lu Z, Kutish GF, Kleiboeker SB, Carrillo C, Zsak L, Rock DL. 1999. An African swine fever virus ORF with similarity to C-type lectins is non-essential for growth in swine macrophages in vitro and for virus virulence in domestic swine. J Gen Virol 80:2693–2697. doi:10.1099/0022-1317-80-10-2693 10573162

[107] Borca MV, Carrillo C, Zsak L, Laegreid WW, Kutish GF, Neilan JG, Burrage TG, Rock DL. 1998. Deletion of a CD2-like gene, 8-DR, from African swine fever virus affects viral infection in domestic swine. J Virol 72:2881–2889. doi:10.1128/JVI.72.4.2881-2889.1998 9525608 PMC109733

[108] Hemmink JD, Khazalwa EM, Abkallo HM, Oduor B, Khayumbi J, Svitek N, Henson SP, Blome S, Keil G, Bishop RP, Steinaa L. 2022. Deletion of the CD2v gene from the genome of ASFV-Kenya-IX-1033 partially reduces virulence and induces protection in pigs. Viruses 14:1917. doi:10.3390/v14091917 36146726 PMC9503863

[109] Borca MV, O’Donnell V, Holinka LG, Risatti GR, Ramirez-Medina E, Vuono EA, Shi J, Pruitt S, Rai A, Silva E, Velazquez-Salinas L, Gladue DP. 2020. Deletion of CD2-like gene from the genome of African swine fever virus strain Georgia does not attenuate virulence in swine. Sci Rep 10:494. doi:10.1038/s41598-020-57455-3 31949276 PMC6965178

[110] Vuono EA, Ramirez-Medina E, Pruitt S, Rai A, Espinoza N, Spinard E, Valladares A, Silva E, Velazquez-Salinas L, Borca MV, Gladue DP. 2022. Deletion of the EP296R gene from the genome of highly virulent African swine fever virus Georgia 2010 does not affect virus replication or virulence in domestic pigs. Viruses 14:1682. doi:10.3390/v14081682 36016304 PMC9415450

[111] Vuono E, Ramirez-Medina E, Silva E, Rai A, Pruitt S, Espinoza N, Valladares A, Velazquez-Salinas L, Gladue DP, Borca MV. 2022. Deletion of the H108R gene reduces virulence of the pandemic Eurasia strain of African swine fever virus with surviving animals being protected against virulent challenge. J Virol 96:e0054522. doi:10.1128/jvi.00545-22 35862691 PMC9327699

[112] Huang L, Liu H, Ye G, Liu X, Chen W, Wang Z, Zhao D, Zhang Z, Feng C, Hu L, Yu H, Zhou S, Zhang X, He X, Zheng J, Bu Z, Li J, Weng C. 2023. Deletion of African swine fever virus (ASFV) H240R gene attenuates the virulence of ASFV by enhancing NLRP3-mediated inflammatory responses. J Virol 97:e0122722. doi:10.1128/jvi.01227-22 36656014 PMC9972963

[113] Ramirez-Medina E, Rai A, Espinoza N, Valladares A, Silva E, Velazquez-Salinas L, Borca MV, Gladue DP. 2023. Deletion of the H240R gene in African swine fever virus partially reduces virus virulence in swine. Viruses 15:1477. doi:10.3390/v15071477 37515164 PMC10384018

[114] Vuono E, Ramirez-Medina E, Pruitt S, Rai A, Silva E, Espinoza N, Zhu J, Velazquez-Salinas L, Gladue DP, Borca MV. 2020. Evaluation in swine of a recombinant Georgia 2010 African swine fever virus lacking the I8L gene. Viruses 13:39. doi:10.3390/v13010039 33383814 PMC7823879

[115] Fan J, Zhang J, Wang F, Miao F, Zhang H, Jiang Y, Qi Y, Zhang Y, Hui L, Zhang D, Yue H, Zhou X, Li Q, Wang Y, Chen T, Hu R. 2024. Identification of L11L and L7L as virulence-related genes in the African swine fever virus genome. Front Microbiol 15:1345236. doi:10.3389/fmicb.2024.1345236 38328426 PMC10848158

[116] Liu Y, Shen Z, Xie Z, Song Y, Li Y, Liang R, Gong L, Di D, Liu J, Liu J, Chen Z, Yu W, Lv L, Zhong Q, Liao X, Tian C, Wang R, Song Q, Wang H, Peng G, Chen H. 2023. African swine fever virus I73R is a critical virulence-related gene: a potential target for attenuation. Proc Natl Acad Sci U S A 120:e2210808120. doi:10.1073/pnas.2210808120 37023125 PMC10104517

[117] Borca MV, Ramirez-Medina E, Silva E, Vuono E, Rai A, Pruitt S, Espinoza N, Velazquez-Salinas L, Gay CG, Gladue DP. 2021. ASFV-G-∆I177L as an effective oral nasal vaccine against the Eurasia strain of Africa swine fever. Viruses 13:765. doi:10.3390/v13050765 33925435 PMC8146859

[118] Tran XH, Le TTP, Nguyen QH, Do TT, Nguyen VD, Gay CG, Borca MV, Gladue DP. 2022. African swine fever virus vaccine candidate ASFV-G-ΔI177L efficiently protects European and native pig breeds against circulating Vietnamese field strain. Trans Bound Emerg Dis 69:e497–e504. doi:10.1111/tbed.14329 34582622

[119] Zhang Y, Ke J, Zhang J, Yue H, Chen T, Li Q, Zhou X, Qi Y, Zhu R, Wang S, Miao F, Zhang S, Li N, Mi L, Yang J, Yang J, Han X, Wang L, Li Y, Hu R. 2021. I267L is neither the virulence- nor the replication-related gene of African swine fever virus and Its deletant is an ideal fluorescent-tagged virulence strain. Viruses 14:53. doi:10.3390/v14010053 35062257 PMC8777747

[120] Ran Y, Li D, Xiong M-G, Liu H-N, Feng T, Shi Z-W, Li Y-H, Wu H-N, Wang S-Y, Zheng H-X, Wang Y-Y. 2022. African swine fever virus I267L acts as an important virulence factor by inhibiting RNA polymerase III-RIG-I-mediated innate immunity. PLoS Pathog 18:e1010270. doi:10.1371/journal.ppat.1010270 35089988 PMC8827485

[121] Vuono EA, Ramirez-Medina E, Pruitt S, Rai A, Espinoza N, Velazquez-Salinas L, Gladue DP, Borca MV. 2021. Evaluation of the function of the ASFV KP177R gene, encoding for structural protein p22, in the process of virus replication and in swine virulence. Viruses 13:986. doi:10.3390/v13060986 34073222 PMC8227490

[122] Sanford B, Holinka LG, O’Donnell V, Krug PW, Carlson J, Alfano M, Carrillo C, Wu P, Lowe A, Risatti GR, Gladue DP, Borca MV. 2016. Deletion of the thymidine kinase gene induces complete attenuation of the Georgia isolate of African swine fever virus. Virus Res 213:165–171. doi:10.1016/j.virusres.2015.12.002 26656424

[123] Moore DM, Zsak L, Neilan JG, Lu Z, Rock DL. 1998. The African swine fever virus thymidine kinase gene is required for efficient replication in swine macrophages and for virulence in swine. J Virol 72:10310–10315. doi:10.1128/JVI.72.12.10310-10315.1998 9811782 PMC110620

[124] Kleiboeker SB, Neilan JG, Kutish GF, Zsak L, Lu Z, Rock DL. 1998. A conserved African swine fever virus right variable region gene, l11L, is non-essential for growth in vitro and virulence in domestic swine. J Gen Virol 79:1189–1195. doi:10.1099/0022-1317-79-5-1189 9603334

[125] Yang J, Zhu R, Zhang Y, Fan J, Zhou X, Yue H, Li Q, Miao F, Chen T, Mi L, Zhang F, Zhang S, Qian A, Hu R. 2023. SY18ΔL60L: a new recombinant live attenuated African swine fever virus with protection against homologous challenge. Front Microbiol 14:1225469. doi:10.3389/fmicb.2023.1225469 37621401 PMC10445127

[126] Borca MV, O’Donnell V, Holinka LG, Ramírez-Medina E, Clark BA, Vuono EA, Berggren K, Alfano M, Carey LB, Richt JA, Risatti GR, Gladue DP. 2018. The L83L ORF of African swine fever virus strain Georgia encodes for a non-essential gene that interacts with the host protein IL-1β. Virus Res 249:116–123. doi:10.1016/j.virusres.2018.03.017 29605728

[127] Liu Y, Li Y, Xie Z, Ao Q, Di D, Yu W, Lv L, Zhong Q, Song Y, Liao X, Song Q, Wang H, Chen H. 2021. Development and in vivo evaluation of MGF100-1R deletion mutant in an African swine fever virus Chinese strain. Vet Microbiol 261:109208. doi:10.1016/j.vetmic.2021.109208 34419775

[128] Ramirez-Medina E, Vuono E, Pruitt S, Rai A, Silva E, Espinoza N, Zhu J, Velazquez-Salinas L, Borca MV, Gladue DP. 2021. Development and in vivo evaluation of a MGF110-1L deletion mutant in African swine fever strain Georgia. Viruses 13:286. doi:10.3390/v13020286 33673255 PMC7918709

[129] Li D, Liu Y, Qi X, Wen Y, Li P, Ma Z, Liu Y, Zheng H, Liu Z. 2021. African swine fever virus MGF-110-9L-deficient mutant has attenuated virulence in pigs. Virol Sin 36:187–195. doi:10.1007/s12250-021-00350-6 33689140 PMC8087726

[130] Wang T, Luo R, Zhang J, Lu Z, Li L-F, Zheng Y-H, Pan L, Lan J, Zhai H, Huang S, Sun Y, Qiu H-J. 2023. The MGF300-2R protein of African swine fever virus is associated with viral pathogenicity by promoting the autophagic degradation of IKKα and IKKβ through the recruitment of TOLLIP. PLoS Pathog 19:e1011580. doi:10.1371/journal.ppat.1011580 37566637 PMC10446188

[131] Wang T, Luo R, Zhang J, Lan J, Lu Z, Zhai H, Li LF, Sun Y, Qiu HJ. 2024. The African swine fever virus MGF300-4L protein is associated with viral pathogenicity by promoting the autophagic degradation of IKKβ and increasing the stability of IκBα. Emerg Microbes Infect 13:2333381. doi:10.1080/22221751.2024.2333381 38501350 PMC11018083

[132] Ramirez-Medina E, Vuono EA, Rai A, Pruitt S, Silva E, Velazquez-Salinas L, Zhu J, Gladue DP, Borca MV. 2020. Evaluation in swine of a recombinant African swine fever virus lacking the MGF-360-1L gene. Viruses 12:1193. doi:10.3390/v12101193 33092258 PMC7589680

[133] Zhang K, Yang B, Shen C, Zhang T, Hao Y, Zhang D, Liu H, Shi X, Li G, Yang J, Li D, Zhu Z, Tian H, Yang F, Ru Y, Cao WJ, Guo J, He J, Zheng H, Liu X. 2022. MGF360-9L is a major virulence factor associated with the African swine fever virus by antagonizing the JAK/STAT signaling pathway. mBio 13:e0233021. doi:10.1128/mbio.02330-21 35076286 PMC8788333

[134] Li D, Peng J, Wu J, Yi J, Wu P, Qi X, Ren J, Peng G, Duan X, Ru Y, Liu H, Tian H, Zheng H. 2023. African swine fever virus MGF-360-10L is a novel and crucial virulence factor that mediates ubiquitination and degradation of JAK1 by recruiting the E3 ubiquitin ligase HERC5. mBio 14:e0060623. doi:10.1128/mbio.00606-23 37417777 PMC10470787

[135] Ramírez-Medina E, Vuono EA, Velazquez-Salinas L, Silva E, Rai A, Pruitt S, Berggren KA, Zhu J, Borca MV, Gladue DP. 2020. The MGF360-16R ORF of African swine fever virus strain Georgia encodes for a nonessential gene that interacts with host proteins SERTAD3 and SDCBP. Viruses 12:60. doi:10.3390/v12010060 31947814 PMC7020080

[136] Li D, Zhang J, Yang W, Li P, Ru Y, Kang W, Li L, Ran Y, Zheng H. 2021. African swine fever virus protein MGF-505-7R promotes virulence and pathogenesis by inhibiting JAK1- and JAK2-mediated signaling. J Biol Chem 297:101190. doi:10.1016/j.jbc.2021.101190 34517008 PMC8526981

[137] Ramirez-Medina E, Velazquez-Salinas L, Rai A, Espinoza N, Valladares A, Silva E, Burton L, Spinard E, Meyers A, Risatti G, Calvelage S, Blome S, Gladue DP, Borca MV. 2023. Evaluation of the deletion of the African swine fever virus gene O174L from the genome of the Georgia isolate. Viruses 15:2134. doi:10.3390/v15102134 37896911 PMC10612027

[138] Ramirez-Medina E, Vuono EA, Pruitt S, Rai A, Espinoza N, Spinard E, Valladares A, Silva E, Velazquez-Salinas L, Borca MV, Gladue DP. 2022. Deletion of an African swine fever virus ATP-dependent RNA helicase QP509L from the highly virulent Georgia 2010 strain does not affect replication or virulence. Viruses 14:2548. doi:10.3390/v14112548 36423157 PMC9694930

[139] Ramirez-Medina E, Vuono E, Pruitt S, Rai A, Silva E, Zhu J, Velazquez-Salinas L, Gladue DP, Borca MV. 2020. X69R is a non-essential gene that, when deleted from African swine fever, does not affect virulence in swine. Viruses 12:918. doi:10.3390/v12090918 32825617 PMC7551905

[140] Sun M, Yu S, Ge H, Wang T, Li Y, Zhou P, Pan L, Han Y, Yang Y, Sun Y, Li S, Li L-F, Qiu H-J. 2022. The A137R protein of African swine fever virus inhibits type I interferon production via the autophagy-mediated lysosomal degradation of TBK1. J Virol 96:e0195721. doi:10.1128/jvi.01957-21 35412346 PMC9093111

[141] Frouco G, Freitas FB, Coelho J, Leitão A, Martins C, Ferreira F. 2017. DNA-binding properties of African swine fever virus pA104R, a histone-like protein involved in viral replication and transcription. J Virol 91:e02498-16. doi:10.1128/JVI.02498-16 28381576 PMC5446646

[142] Gaudreault NN, Richt JA. 2019. Subunit vaccine approaches for African swine fever virus. Vaccines (Basel) 7:56. doi:10.3390/vaccines7020056 31242632 PMC6631172

